# EDECO: An Enhanced Educational Competition Optimizer for Numerical Optimization Problems

**DOI:** 10.3390/biomimetics10030176

**Published:** 2025-03-12

**Authors:** Wenkai Tang, Shangqing Shi, Zengtong Lu, Mengying Lin, Hao Cheng

**Affiliations:** 1 School of Computer Science and Information Security, Guilin University of Electronic Technology, Guilin 541000, China; 2300310424@mails.guet.edu.cn (W.T.); chenghao@guet.edu.cn (H.C.); 2 School of Information Science and Engineering, Southeast University, Nanjing 210096, China; sqshi@seu.edu.cn; 3 Ruijie Networks Co., Ltd., Fuzhou 350000, China

**Keywords:** metaheuristic, educational competition optimizer, engineering optimization, CEC 2017 test suite

## Abstract

The Educational Competition Optimizer (ECO) is a newly proposed human-based metaheuristic algorithm. It derives from the phenomenon of educational competition in society with good performance. However, the basic ECO is constrained by its limited exploitation and exploration abilities when tackling complex optimization problems and exhibits the drawbacks of premature convergence and diminished population diversity. To this end, this paper proposes an enhanced educational competition optimizer, named EDECO, by incorporating estimation of distribution algorithm and replacing some of the best individual(s) using a dynamic fitness distance balancing strategy. On the one hand, the estimation of distribution algorithm enhances the global exploration ability and improves the population quality by establishing a probabilistic model based on the dominant individuals provided by EDECO, which solves the problem that the algorithm is unable to search the neighborhood of the optimal solution. On the other hand, the dynamic fitness distance balancing strategy increases the convergence speed of the algorithm and balances the exploitation and exploration through an adaptive mechanism. Finally, this paper conducts experiments on the proposed EDECO algorithm with 29 CEC 2017 benchmark functions and compares EDECO with four basic algorithms as well as four advanced improved algorithms. The results show that EDECO indeed achieves significant improvements compared to the basic ECO and other compared algorithms, and performs noticeably better than its competitors. Next, this study applies EDECO to 10 engineering constrained optimization problems, and the experimental results show the significant superiority of EDECO in solving real engineering optimization problems. These findings further support the effectiveness and usefulness of our proposed algorithm in solving complex engineering optimization challenges.

## 1. Introduction

The optimization problem is an important challenge that we need to face in various scientific and engineering disciplines. These problems require identifying the most suitable solution from a large number of possible options, according to particular objective functions and under specific constraints [1]. The inherent characteristics of optimization problems often include high dimensionality, non-linearity, multimodality, and complexity, making them difficult to tackle with conventional approaches [2,3]. Traditional optimization methods, such as gradient based techniques and linear programming, have been widely used in the past [4]. However, they face significant limitations when dealing with complex optimization problems. Gradient-based methods, for instance, require the objective function to be differentiable and continuous, and they are prone to getting stuck in local optima and unable to jump out, especially in multimodal optimization landscapes [5]. Linear programming is effective for linear problems but struggles with the nonlinear relationships that are common in many real-world scenarios. As a result, there is an urgent need for more powerful and flexible optimization tools. Over recent years, continuously growing metaheuristic algorithms have become increasingly important in solving various types of complex optimizations [6]. These algorithms are inspired by animal behavior, evolution of species and physical processes in nature. They have several distinct benefits over traditional methods. First, MAs are usually more robust and less sensitive to problem characteristics. They can effectively handle nonlinear, nondifferentiable and discontinuous objective functions [7]. Second, due to the use of stochastic and population search mechanisms, MAs have a strong ability to explore the search space and avoid local optima. This enables them to find high-quality solutions in a wider solution space. In addition, MAs are usually easy to implement and can be adapted to different problem domains with relative ease [8].

The applications of MAs are wide and varied. In the field of engineering design, they have been used to optimize the parameters of mechanical structures [9], circuits [10], and communication systems [11], thereby improving performance and cost-effectiveness. In the field of machine learning, metaheuristic algorithms have been used for feature selection [12], neural network training [13], and hyperparameter tuning [14], resulting in improved model accuracy and efficiency [15]. In addition, they have been applied to optimization problems such as task planning [16], image segmentation [17] and cloud resource scheduling [18]. MAs can be categorized into two main types based on the number of solutions generated in each iteration: single-solution-based algorithms and population-based algorithms [19].

In single-solution-based algorithms, only a single individual is utilized to search the solution space. Single-solution-based algorithms relies on iterative improvement to progressively refine the initial solution. Such algorithms aim to find optimal or near-optimal solutions by exploring and exploiting the neighborhood of the current candidate solution, e.g., Taboo Search (TS) [20], Large Neighborhood Search (LNS) [21], and Non-monopolize Search (NS) [22].

Population-based algorithms derived their inspiration from a variety of behaviors in nature, including animals and plants engaging in foraging, mating, and pollination. It can be further categorized into evolutionary-based algorithms (EbA), swarm-based algorithm, physics-based algorithm (PbA), mathematical-based algorithm (MbA), and human-based algorithms (HbA) [23]. Evolutionary-based algorithms are a class of optimization and search algorithms that impersonate the process of natural selection and evolution observed in biological systems. These algorithms mimic genetic variation, selection, and reproduction mechanisms to iteratively evolve a population of candidate solutions toward optimal or near-optimal solutions for a given problem. In the 1970s, Genetic Algorithm (GA) gained popularity with Holland’s work, relying on efficient encoding, decoding, and proper parameter configuration [24]. Other algorithms in this category also include Differential Evolution (DE) [25], Evolutionary Strategies (ES) [26], Alpha evolution (AE) [27] and Evolutionary Mating Algorithm (EMA) [28]. Swarm-based algorithms, which emulate the natural behaviors observed in various biological entities, are characterized by their decentralized control and inherent self-organizational capabilities. Their versatility in different fields, effective utilization of solution space information, and fewer parameters to tune reassure the audience about their wide applicability. Particle Swarm Optimization (PSO) is one of the classical instances in which the foraging behavioral patterns of bird flocks are simulated to locate the ideal solution [29]. Other prominent algorithms in this category comprise Artificial Bee Colony (ABC) [30], Crayfish Optimization Algorithm (COA) [31], Secretary bird optimization algorithm (SBOA) [32], Remora Optimization Algorithm (ROA) [33], Tuna Swarm Optimization (TSO) [34] and Sea Horse Optimizer (SHO) [35]. Physics-based algorithms are nature-inspired optimization techniques that imitate physical and chemical law phenomena inspired by electromagnetic forces, gravity, inertia, river systems, chemical changes, and other natural phenomena. Gravitational Search Algorithm (GSA) is the best example of the applicability of physics-based algorithms which simulate mass interactions based on the law of gravity [36]. Other notable physics-based algorithms include Multi-Verse Optimizer (MVO) [37], Kepler optimization algorithm (KOA) [38], Fick’s Law Algorithm (FLA) [39], Polar Lights Optimization (PLO) [40], Equilibrium Optimizer (EO) [41], Newton-Raphson-based Optimizer (NRBO) [42] and Snow Ablation Optimizer (SAO) [43]. Mathematical-based algorithms are inspired by mathematical theories, functions, and formulas, which have demonstrated significant promise in enhancing the computing efficacy of optimization approaches. One of the noteworthy methods in this category is the Sine Cosine Algorithm (SCA), which applies the concept of trigonometric functions to create an algorithmic model [44]. Generalized Normal Distribution Optimization (GNDO) [45], Gold Sine Algorithm (GSA) [46], Quadratic Interpolation Optimization (QIO) [47], Quasi-Random Fractal Search (QRFS) [48], Four Vector Intelligent Metaheuristic (FVIM) [49] and Exponential Distribution Optimizer (EDO) [50] are other instances in this category. Human-based algorithms have drawn inspiration from human societal behaviors, such as political campaigns, competition, learning, and cultural influence. The examples of human-based algorithms are Teaching-Learning-Based Optimization (TLBO) [51], Group Teaching Optimization Algorithm (GTOA) [52], Catch Fish Optimization Algorithm (CFOA) [53], Preschool Education Optimization Algorithm (PEOA) [54], Hiking Optimization Algorithm (HOA) [55] and Student Psychology Based Optimization (SPBO) [56].

Educational Competition Optimizer (ECO) is a new human-based MAs proposed by Lian et al. in 2024 [57]. The algorithm divides the population into two groups: school population and student population, and uses a roulette-based iterative framework to guide the search process. In initial studies, ECO demonstrated strong convergence and superior search capabilities, achieving the best performance on the CEC 2021 benchmark suite. However, by analyzing the updating method and results in test functions of ECO, it is found that it faces some limitations, such as reduced population diversity, low convergence accuracy, and the tendency to converge to a local optimum when solving complex optimization problems. In order to improve ECO, some suitable strategies must be chosen to overcome these limitations. Generally, improving the existing one and hybridizing different MAs are two enhancing strategies. In the improvement process, one or more methods are introduced in the existing MAs to obtain better results.

In this work, an enhanced educational competition optimizer is proposed with an improved core containing the above two enhancing strategies. Firstly, we combine the basic ECO and the estimation of distribution algorithm (EDA) to ultimately enhance the global search capability, improve the population quality, and address the problem of the algorithm’s inability to search for the neighborhood of the optimal solution through modeling the EDA’s probability distribution with the ECO’s dominant population as well as replacing the ECO’s population with the EDA’s sampling. In addition, we propose a dynamic fitness–distance-balanced strategy (DFS) to balance the exploitation and exploration of ECO for its own deficiency, which improves the convergence speed of the algorithm through an adaptive mechanism. In summary, the work presented and contributions made in this paper are shown below.

(1) We propose a hybrid search framework to combine ECO and EDA, which effectively improves the performance.
(2) We propose a dynamic fitness distance balancing strategy, which promotes convergence accuracy and balances the exploitation and exploration capabilities.
(3) We conduct a series of numerical experiments using the CEC2017 test suite to verify the superiority of the improved strategy and the proposed EDECO.
(4) We further evaluate the performance and potential of EDECO in real-world applications using ten engineering constrained optimization problems.

The organization of this paper is outlined as follows: Section 2 offers an overview of the foundational principles and mathematical formulation underlying the basic ECO. Section 3 elaborates on the core ideas behind the proposed EDECO, accompanied by detailed pseudo-code, flowcharts, and an in-depth analysis of its computational complexity. Section 4 evaluates the optimization capabilities of EDECO using the CEC2017 benchmark suites, while also examining the contributions of each enhancement strategy to its overall performance. Section 4 also applies EDECO to ten engineering constrained optimization problems. Finally, Section 5 summarizes the principal findings of this research and highlights potential directions for future research.

## 2. Educational Competition Optimizer (ECO)

The ECO is designed to simulate competitive strategies at the elementary, middle, and high school levels. As competitive pressures increase and the number of schools decreases, the optimization process of ECO is divided into three successive phases. These phases achieve a smooth transition from exploration to exploitation. The mathematical model of ECO is representing as follows.

At this stage, schools determine their best educational location based on the average location of their population. In turn, students compete by aiming for proximity to the nearest school. This approach reflects the initial stage of exploration, where schools and students search for a favorable location without many constraints.

Middle School Stage: During this phase, the number of schools declined, leading them to assess their location based on the average location of the population and the most recognizable locations. Students compete by targeting the nearest school, reinforcing the concept of proximity. This phase introduced a balance between exploration and utilization, and schools became more insightful in their location strategies.

High School Stage: At this stage, the school implements a more refined assessment process that considers the average, best and worst position of the population to determine their educational location. With only one school to choose from, students strive to compete for this single goal, emphasizing proximity. This phase represents the transition to the development phase, where strategic precision is critical and competition becomes more intense.

### 2.1. Population Initialization

ECO, as a meta-heuristic algorithm, has a different method of population initialization than other algorithms. It utilizes logical chaos mapping to generate initial populations to model social disorder due to lack of education. In this paper, the number of populations is N and the upper and lower boundaries of the problem space are Ub and Lb. The formula for the logical chaos mapping is shown below.

(1) $$x_{i}=\alpha \times x_{i-1}\times \left(1-x_{i-1}\right),0\leq x_{0}\leq 1,i=1,\;2,\ldots ,N$$

where $x_{i}$ represents the $i^{th}$ value and $x_{i-1}$ represents the previous value. The parameter α is defined as a constant, with its value set to 4 in this work. The chaotic value $x_{i}$ can be mapped to the search space:

(2) $$X_{i}=Lb+\left(Ub-Lb\right)\times x_{i}$$

### 2.2. Primary School Stage

ECO divides populations into school population and student population at the primary level. The top 20% of individuals ranked in terms of fitness comprise the school population and the rest comprise the student population. At this stage, individuals in the school population utilize the average position of the population to adjust their position, and individuals in the student population decide the direction of movement based on proximity to the school population. Equations (4) and (5) provide the updating methods for the school population and the student population, respectively.

(3) $$\omega =0.1\times \ln\left(2-\frac{t}{t_{\max}}\right)$$

(4) $$School:X_{i}^{t+1}=X_{i}^{t}+\omega \times \left(X_{imean}^{t}-X_{i}^{t}\right)\times Levy\left(D\right)$$

(5) $$Students:X_{i}^{t+1}=X_{i}^{t}+\omega \times \left(close\left(X_{i}^{t}\right)-X_{i}^{t}\right)\times randn$$

where t and $t_{\max}$ denote the current iteration number and the maximum iteration number. $X_{i}^{t}$ represents the current position and $X_{i}^{t+1}$ denotes the position after the next update. $X_{imean}^{t}$ represents the average position of the vector for the $i^{th}$ school agent in the $t^{th}$ iteration. $Levy\left(D\right)$ is a random vector that follows the Lévy distribution. $close\left(X_{i}^{t}\right)$ indicates the location of the school closest to $X_{i}^{t}$. randn represents a random variable following a normal distribution.

### 2.3. Middle School Stage

At the middle school stage, ECO selects the top 10 percent of individuals in terms of fitness to form the school population and the remainder to form the student population. Unlike the primary stage, where ECO utilizes the average and optimal positions to move the school population, the student population is divided into two groups based on their academic potential to move their positions separately. Thus, the mathematical formulation of the school population and the student population is shown below.

(6) $$School:X_{i}^{t+1}=X_{i}^{t}+\left(X_{best}^{t}-X_{mean}^{t}\right)\times e^{\left(\frac{t}{t_{\max}}-1\right)}\times Levy\left(D\right)$$

(7) $$Students:X_{i}^{t+1}=X_{i}^{t}-\omega \times close\left(X_{i}^{t}\right)-P\times \left(E\times \omega \times close\left(X_{i}^{t}\right)-X_{i}^{t}\right)$$

(8) $$P=4\times randn\times \left(1-\frac{t}{t_{\max}}\right)$$

(9) $$E=\left\{\begin{array}{l} \frac{\pi }{P}\times \frac{t}{t_{\max}},R_{t}>H\\ 1,R_{t}\leq H \end{array}\right.$$

where $X_{best}^{t}$ denotes the position of the best school agent from now on. $X_{mean}^{t}$ represents the average position of whole agents the $t^{th}$ iteration. The talent values of different students are simulated using the random number $R_{t}$, which takes on a value within the range of [0, 1]. The parameter H is set to 0.5.

### 2.4. High School Stage

The grouping of populations at the high school stage is the same as at the middle school stage. The school population achieves better search by referring to the average position, the optimal position. Student populations move closer to the optimal position.

(10) $$School:X_{i}^{t+1}=X_{i}^{t}+\left(X_{best}^{t}-X_{mean}^{t}\right)\times randn-\left(X_{best}^{t}-X_{mean}^{t}\right)\times randn$$

(11) $$Students:X_{i}^{t+1}=X_{i}^{t}-P\times \left(E\times X_{best}^{t}-X_{i}^{t}\right)$$

The pseudo-code of ECO is represented as shown in Algorithm 1.
**Algorithm 1**: Pseudo-code of the ECO1: Initialize the ECO parameters2: Initialize the solutions’ positions randomly Equation (2)3: **While** *t* < *t*_max_4: Calculate the fitness function5: Find the best position and worst position6: Calculate *R_t_*, *P*, *E*7: **For**
*i* = 1: *N* **do**8: Stage 1: Primary school competition9:  **If** mod (*t*, 3) == 1 **Then**10:   Update school position by Equation (4)11:   Update student position by Equation (5)12:  **End if**13: Stage 2: middle school competition14:  **If** mod (*t*, 3) == 2 **Then**15:   Update school position by Equation (6)16:   Update student position by Equation (7)17:  **End if**18: Stage 3: High school competition19:  **If** mod (*t*, 3) == 3 **Then**20:   Update school position by Equation (10)21:   Update student position by Equation (11)22:  **End if**23: **End for**
24: *t* = *t* + 124: **End while**
25: Return the best solution and fitness

## 3. Enhanced Educational Competition Optimizer

Although ECO shows promising prospects as a metaheuristic algorithm, it faces significant challenges in complex optimization scenarios. The ECO exhibits inherent limitations, including difficulties in balancing exploitation and exploration, and a tendency to converge to local optima. These weaknesses hinder its effectiveness in dealing with complex and demanding optimization problems. To address these restrictions, this paper proposes the Enhanced Educational Competition Optimizer (EDECO), which improves performance through hybrid estimation of distribution algorithm and the introduction of a dynamic fitness distance balancing strategy. In this section, the improved techniques of the proposed EDECO are first described in detail. Subsequently, pseudo-code and flowcharts are presented. Finally, this section analyzes the time complexity of the proposed EDECO algorithm.

### 3.1. Hybrid Search Framework for EDA

**Figure 1:**
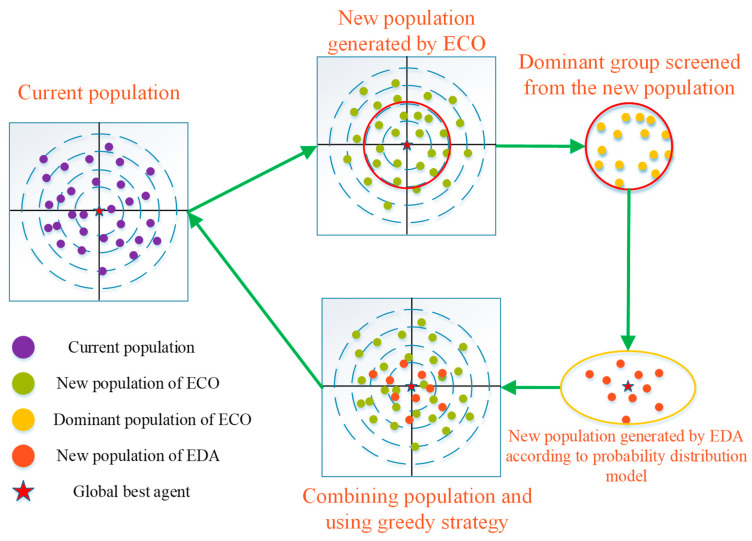
Sketch for hybrid search framework.

The quality of the ECO’s population decreases progressively with the depth of the search, and its global exploration capability is limited. To solve this concern, this paper proposes a hybrid search framework to incorporate the EDA method. Unlike other hybrid algorithms, the novelty of this work is reflected in the harmonious synergy between the ECO and EDA methods. Common hybrid algorithms are embedding multiple algorithms into different search stages or reusing multiple algorithms for the same population. The hybrid search framework proposed in this paper realizes a new hybrid technique, and the process is shown in Figure 1.

Specifically, the solution space is first searched using the ECO method to generate new populations. Then, some individuals with better fitness values are screened from the new population to form a dominant group, and a probability distribution model is constructed. The EDA method generates new individuals by sampling according to this probability distribution model. The population that proceeds to the next iteration is filtered from these individuals and the individuals generated by ECO through a greedy strategy. On the one hand, the dominant population that creates the probabilistic model is composed of both ECO and EDA individuals, which mitigates the shortcoming of orthogonality in the evolutionary direction of EDA. On the other hand, EDA utilizes the powerful exploration ability to boost the population diversity of ECO, which compensates for its inability to search the optimal solution domain. The application of greedy strategy accelerates the convergence speed of EDECO. The EDA algorithm contains three steps: selecting the dominant individuals, modeling the probability distribution, and sampling to generate new individuals. In this paper, a Gaussian probability model is used. The joint Gaussian probability density function for a random vector X with D dimensions can be parameterized by the mean μ and the covariance matrix C as follows.

(12) $$G{\left(X\right)}_{\left(\mu ,C\right)}=\sqrt{\frac{1}{{\left(2\pi \right)}^{D}\det\left(C\right)}}\times e^{\left(\frac{-{\left(X-\mu \right)}^{T}\times C^{-1}\times \left(X-\mu \right)}{2}\right)}$$

(13) $$\mu =\frac{1}{N_{d}}\times \sum_{do=1}^{N_{d}}X_{do}$$

(14) $$C=\frac{1}{N_{d}}\times \sum_{i=1}^{N_{d}}\left(X_{i}-\mu \right)\times {\left(X_{i}-\mu \right)}^{T}$$

where $N_{d}$ is the number of dominant individuals selected from the new population generated by ECO. $X_{do}$ denotes the $do^{th}$ dominant individual with better fitness. The EDA generates new individuals based on a Gaussian probability distribution model with mean μ and covariance matrix C as follows.

(15) $$X_{i}^{t+1}=\mu +g,g∼N\left(0,C\right)$$

### 3.2. Dynamic Fitness Distance Balancing Strategy

ECO exhibits a weak exploitation in the later stages of the search, limiting further accurate search of the optimal solution domain. To address this issue, this paper uses a dynamic fitness distance balancing strategy (DFS) to modify the update strategy at the high school level. The DFS is leveraged to select the optimal individual $X_{DFS}$ to guide the student population at the high school stage towards the optimal position, and to achieve a relative balance between exploitation and exploration through adaptive parameters. The DFS strategy is an adaptive variant of the fitness distance balancing strategy. The basic fitness distance balancing strategy selects the optimal solution based on a weighted score of fitness and distance. When considering the two metrics, distance and fitness, it is assumed that both contribute to the search equally. However, for metaheuristic algorithms, the proper choice of exploitation or exploration at the suitable time is significant. Algorithms need to avoid local optima through strong exploration capability and need intensive exploitation ability to search accurately. Thus, it is necessary to implement an adaptive mechanism to dynamically adjust the weights of fitness and distance. The details of the DFS strategy are described below.

(16) $$S_{i}=\omega \times norm\left(Fit_{i}\right)+\left(1-\omega \right)\times norm\left(Dis_{i}\right)$$

(17) $$Dis_{i}=\sqrt{{\left(X_{i,1}-X_{best,1}\right)}^{2},{\left(X_{i,2}-X_{best,2}\right)}^{2}+\cdots +{\left(X_{i,D}-X_{best,D}\right)}^{2}}$$

(18) $$\omega =\frac{\mathrm{mod}\left(\frac{t_{\max}}{\alpha },t\right)}{t_{\max}}\times \left(1-\beta \right)+\beta$$

where $S_{i}$ is the DFS score of the $i^{th}$ individual, weighted by the normalized value $norm\left(Fit_{i}\right)$ of fitness and the normalized value $norm\left(Dis_{i}\right)$ of distance for this individual. Before the start of each iteration, $X_{DFS}^{t}$ is selected based on the scores of each individual. Thus, Equation (7) can be modified as follows.

(19) $$Students:X_{i}^{t+1}=X_{DFS}^{t}-\omega \times close\left(X_{i}^{t}\right)-P\times \left(E\times \omega \times close\left(X_{i}^{t}\right)-X_{i}^{t}\right)$$

### 3.3. The Framework of EDECO

**Figure 2:**
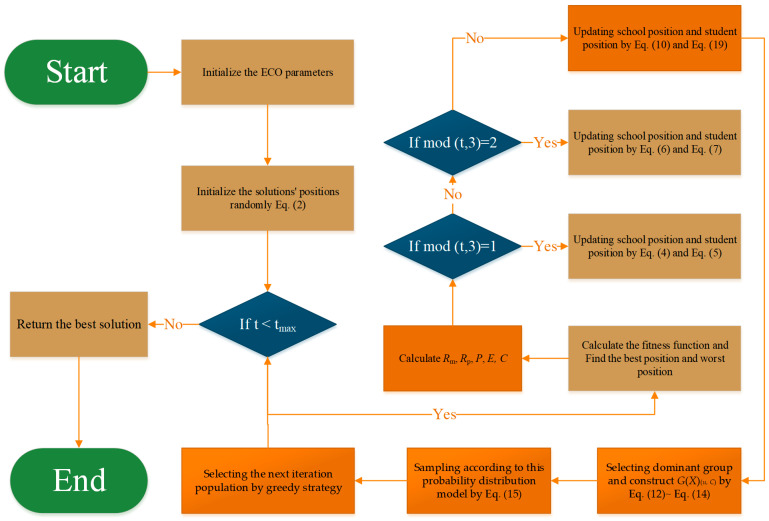
A flowchart of the proposed EDECO.

This study proposes EDECO, which combines ECO with the above two mechanisms. The pseudo-code of the proposed EDECO algorithm is shown in Algorithm 2. In each iteration, EDECO first generates new individuals using the ECO method and then samples new individuals using the EDA method, and the new individuals of both methods are selected by a greedy strategy to select N individuals for the next iteration. The flowchart of the algorithm is shown in Figure 2.
**Algorithm 2**: Pseudo-code of the EDECO1: Initialize the ECO parameters2: Initialize the solutions’ positions randomly Equation (2)3: **While** *t* < *t*_max_4: Calculate the fitness function5: Find the best position and worst position6: Calculating *R_t_*, *P*, *E*, *C*, *S*7: **For**
*i* = 1: *N* **do**8: Stage 1: Primary school competition9:  **If** mod (*t*, 3) == 1 **Then**10:   Updating school position by Equation (4)11:   Updating student position by Equation (5)12:  **End if**13: Stage 2: middle school competition14:  **If** mod (*t*, 3) == 2 **Then**15:   Updating school position by Equation (6)16:   Updating student position by Equation (7)17:  **End if**18: Stage 3: High school competition19:  **If** mod (*t*, 3) == 3 **Then**20:   Updating school position by Equation (10)21:   Updating student position by Equation (19)22:  **End if**23: **End for**
24: Selecting dominant group and construct *G*(*X*)_(*u, C*)_ by Equations (12)–(14)25: Sampling according to this probability distribution model by Equation (15)26: Selecting the next iteration population by greedy strategy27: *t* = *t* + 128: **End while**
29: Return the best solution

### 3.4. Computational Complexity Analysis

In this paper, the EDECO algorithm is obtained by combining EDA and DFS. From Algorithm 2 and Figure 2, EDECO contains population initialization, fitness calculation, generation of new individuals by ECO method and generation of new individuals by EDA method. We assume that N is the number of search agents, D is the problem size, and the maximum number of iterations is T. Then, the time complexity of initializing the populations is $O\left(N\times D\right)$. At each iteration, the ECO method updates N individuals, and the EDA method updates $N_{E}$ individuals, so the time complexity of the ECO method is $O\left(T\times N\times D\right)$, and the time complexity of the EDA method is $O\left(T\times N_{E}\times D\right)$. Since the DFS strategy is a selection strategy that does not generate additional individuals, hence, DFS does not increase the time complexity in the whole process. In conclusion, the ECO is approximately $O\left(T\times N\times D\right)$ and the EDECO is approximately $O\left(T\times \left(N+N_{E}\right)\times D\right)$. EDECO has a higher time complexity than basic ECO due to the existence of the EDA phase. However, in this paper, the maximum number of function evaluations is used as a stopping criterion to achieve a fair comparison.

## 4. Numerical Experiment and Analysis

Since the original paper has already thoroughly compared the ECO algorithms against various algorithms using the CEC 2021 benchmark function, we focused on more challenging optimization scenarios for our study. Specifically, we validate the proposed EDECO algorithm through three experiments. A total of 29 CEC2017 benchmark test functions and 10 engineering constrained optimization problems were utilized to check the performance of EDECO. Section 4.1 contains comprehensive details of the CEC2017 test suite and parameter configurations of the compared algorithms. In Section 4.2, we discuss the impact of each improvement strategy on EDECO. Section 4.3 shows the results of comparing EDECO with the basic algorithm and the improved algorithms. Section 4.4 confirms the ability of EDECO to solve constrained optimization problems.

### 4.1. Experiment Setting and Evaluation Criteria

The proposed EDECO algorithm has been implemented in MATLAB R2023a on a computer system equipped with an AMD R9 7940HX processor running at 2.130 GHz and 32 GB of RAM.

The CEC test function suite is a widely recognized and authoritative benchmark suite in the field of computational intelligence, particularly for evaluating the performance of optimization algorithms. Introduced by the IEEE Congress on Evolutionary Computation (CEC), these test functions are carefully designed to cover a wide range of optimization challenges, including unimodal, multimodal, separable, non-separable, and rotated problems. This study employs the CEC2017 benchmark suite (30-dimensional, 50-dimensional, and 100-dimensional). Detailed parameter configurations of the CEC2017 test suite are presented in Table 1. In this work, the maximum number of function evaluations ($Fes_{\max}$) is used instead of the maximum number of iterations as a stopping criterion to fairly compare the performance of each algorithm. The $Fes_{\max}$ is 5000 times the problem dimensions, i.e., 50,000 (10D), 150,000 (30D), 250,000 (50D), and 500,000 (100D). All experimental results were obtained by running each algorithm independently 30 times.

In order to fully demonstrate the superiority of EDECO, two derived algorithms and eight advanced algorithms were chosen for comparison with EDECO. The derived algorithms were designed to perform ablation experiments and include: EECO and DECO. The advanced competitors include the basic algorithms: SAO [43], CFOA [53], DBO [58], and MRFO [59], as well as the improved algorithms: ISGTOA [52], EMTLBO [60], TERIME [61], and AFDB-ARO [62]. The detailed parameter settings for the relevant algorithms are provided in Table 2.

### 4.2. Analysis of the Performance of Each Strategy on EDECO

As mentioned earlier, the proposed EDECO incorporates two improvement strategies: the hybrid search framework (HSF) and the dynamic fitness distance balancing strategy (DFS). The purpose of the experiments in this subsection is to examine the impact of these added optimization mechanisms on the performance of the basic EDECO. Therefore, in this study, the ECO integrated with HSF (EECO) and the ECO integrated with DFS (DECO) are subjected to ablation experiments with EDECO as well as the basic ECO under the CEC2017 benchmark functions. The parameter settings for these ECO variants and EDECO are the same. These results are reported using the nonparametric Wilcoxon rank sum test with a significance level of 0.05 and the Friedman test as shown in Table 3 and Table 4.

**Figure 3:**
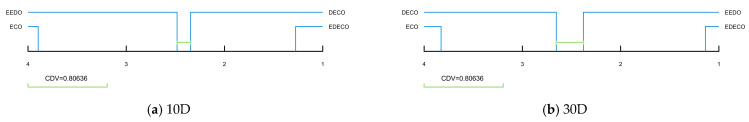

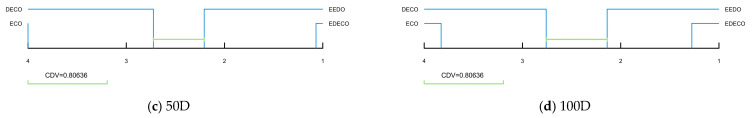
Multiple comparisons using CDV to evaluate ECO, EDECO and its two variants.

Based on the *p*-values in the last column of Table 3, we can know that there are performance differences between these four algorithms. Specifically, EDECO, which incorporates two strategies, achieves the first place in all four cases with rankings of 1.28, 1.14, 1.07 and 1.28, EECO ranks second on 30D,50D and 100D, DECO ranks second on 10D, and ECO is at the bottom of the list in all cases. Based on the rankings of EECO and EDCO, we can see that the HSF strategy enhances ECO more than the DFS enhances the performance of ECO. Table 4 records the wins and losses of EDECO, EECO, DECO and basic ECO. We can observe that the number of functions in which EDECO significantly outperforms ECO is the largest, with EECO and EDCO decreasing in that order, which matches the results of the Friedman test. Furthermore, we used the Nemenyi test as a post hoc test to further demonstrate the differences between EDECO, EECO, DECO, and ECO based on Friedman rankings. Figure 3 presents the magnitude of differences among ECO, EDECO and its two variants, in which the algorithms with no significant difference in terms of performance can be connected using CDV.

When the ranking difference between the two algorithms is less than the CDV, then the difference between the two algorithms is considered insignificant, and vice versa, the difference is significant. As shown in Figure 3, EDECO significantly outperforms ECO and the two variants, and the two variants similarly significantly outperform ECO. This indicates that a single improvement mechanism significantly improves the performance of ECO. The greatest degree of ECO performance improvement occurs when the two mechanisms are combined. Therefore, we can conclude that the proposed improvement strategies significantly enhance the performance of ECO and EDECO is a promising ECO variant.

### 4.3. Analysis of the Results of CEC2017 Test Suite

**Figure 4:**
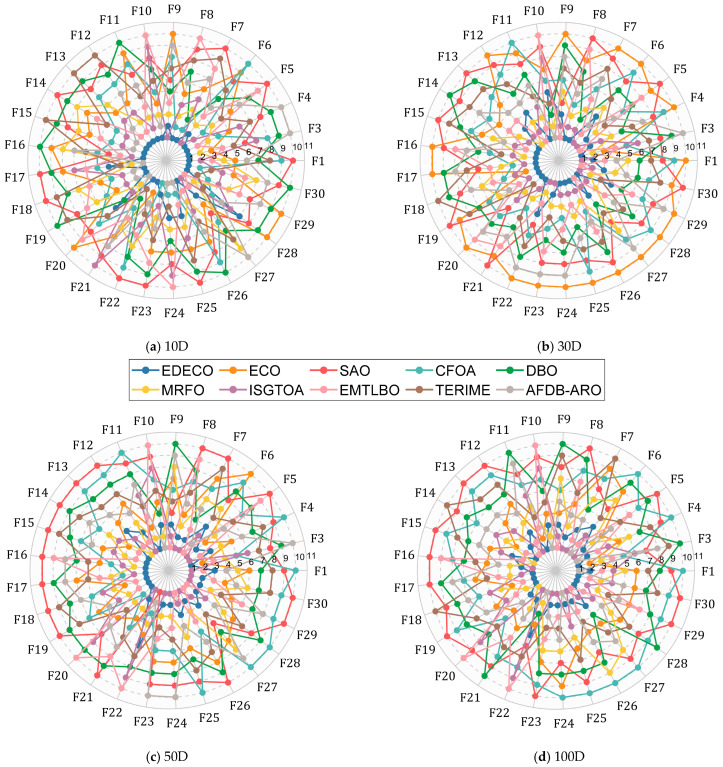
Rankings based on “Ave” of EDECO and its comparison algorithms on each benchmark function.

In this subsection, the performance of the proposed EDECO is evaluated using the CEC2017 test suite and eight advanced basic and improved algorithms are compared including the basic algorithms: SAO, CFOA, DBO, and MRFO, as well as the improved algorithms: ISGTOA, EMTLBO, TERIME, and AFDB-ARO. To ensure a fair comparison, the maximum number of function evaluations is employed as a stopping criterion for all the experiments in this subsection, and the parameter settings for all the algorithms follow the recommended values from the source literature. Table A1, Table A2, Table A3 and Table A4 in Appendix A provide detailed results obtained by EDECO and the compared algorithms, including the optimal value, average value, standard deviation and ranking. Here, the performance of EDECO and competitors on each function is visualized initially through radar plots, as shown in Figure 4. In the radar plot, the ranking of each algorithm on each function is connected into a polygon whose area size reflects how much better or worse the algorithm performs. The smaller the area, the better the performance of this algorithm. Based on Figure 4, we can roughly conclude that EDECO achieves the best overall results in all dimensions and outperforms the comparison algorithms. In the following, the experimental results of EDECO and the comparison algorithms will be further analyzed using statistical methods to avoid experimental bias caused by relying only on the average value.

**Figure 5:**
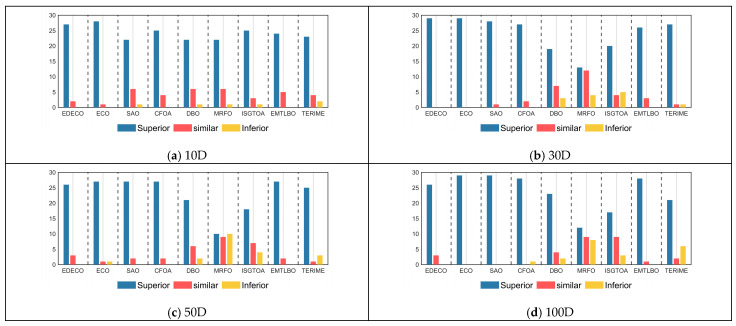
The visualization of Wilcoxon rank sum test results between EDECO and its competitors (a = 0.05).

Table 5 summarizes the results of Wilcoxon rank sum test for 30 independent results obtained by EDECO, ECO and eight competitors with significance level a = 0.05. In Table 5, the symbols “+/=/−” mean that EDECO is superior/similar/inferior to its competitors in terms of performance, respectively. The winners and losers of EDECO and its competitors are visualized in Figure 4. According to Figure 5 and Table 5, we observe that the total number of “+” obtained by the proposed EDECO in each dimension is more than “−”, which indicates that EDECO significantly outperforms both ECO and the competitors in terms of overall performance. Details of the analysis are given below.

For 10D, EDECO is superior (inferior) to SAO, CFOA, DBO, MRFO, ISGTOA, EMTLBO, TERIME, AFDB-ARO on 27(0), 28(0), 22(1), 25(0), 22(1), 22(1), 25(1), 24(0) and 23(2) test functions. That is, EDECO beats all competitors on 10D.

For 30D, EDECO is superior (inferior) to SAO, CFOA, DBO, MRFO, ISGTOA, EMTLBO, TERIME, AFDB-ARO on 29(0), 29(0), 28(0), 27(0), 19(3), 13(4), 20(5), 26(0) and 27(1) test functions. That is, EDECO beats all competitors on 30D.

For 50D, EDECO is superior (inferior) to SAO, CFOA, DBO, MRFO, ISGTOA, EMTLBO, TERIME, AFDB-ARO on 26(0), 27(1), 27(0), 27(0), 21(2), 10(10), 18(4), 27(0) and 25(3) test functions. That is, EDECO beats all competitors except ISGTOA on 50D.

For 100D, EDECO is superior (inferior) to SAO, CFOA, DBO, MRFO, ISGTOA, EMTLBO, TERIME, AFDB-ARO on 26(0), 29(0), 29(0), 28(1), 23(2), 12(8), 17(3), 28(0) and 21(6) test functions. That is, EDECO beats all competitors on 100D.

According to the “Total” in the last column of Table 5, EDECO is superior (inferior) to SAO, CFOA, DBO, MRFO, ISGTOA, EMTLBO, TERIME, AFDB-ARO on 108(0), 113(0), 106(1), 107(0), 85(8), 57(23), 80(13), 105(0) and 96(12) functions. That is to say, compared to the four basic algorithms and the 4 improved algorithms, EDECO has more “+” than “−”, which implies that EDECO has the best performance. Thus, we can conclude that the proposed EDECO has the best overall performance compared to SAO, CFOA, DBO, MRFO, ISGTOA, EMTLBO, TERIME, AFDB-ARO.

**Figure 6:**
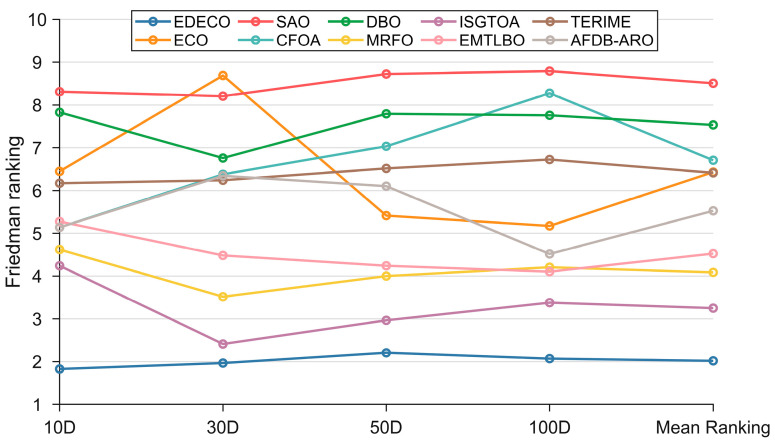
The visualization of Friedman test results between EDECO and its competitors.

The Freidman test was next used to compare the difference in performance between EDECO and its competitors. The rankings and *p*-values obtained through the Friedman test are summarized in Table 6 and visually depicted in Figure 6. According to Table 6, all *p*-values are less than 0.05, which indicates that there is a performance difference between EDECO and ECO as well as the other competitors on all dimensions of the CEC2017 test suite. Figure 6 shows that EDECO achieves the smallest Friedman rankings of 1.8276, 1.9655, 2.2069, and 2.0690, respectively. That is, EDECO outperforms all the competing algorithms on all dimensions, which is consistent with the analysis results of the Wilcoxon rank sum test. Furthermore, EDECO’s ranking does not float significantly with the change in dimensions, showing its superior stability and scalability.

**Figure 7:**
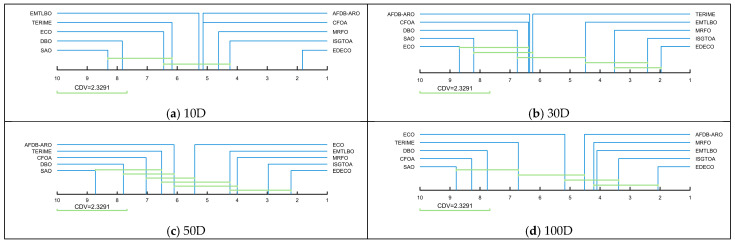
Multiple comparisons using CDV to evaluate EDECO and its competitors.

Based on the Friedman test, the Nemenyi test was used to further quantify the magnitude of the difference between EDECO and its competitors. In the Nemenyi test, q is obtained by querying a statistical table of the F distribution. The critical difference value (CDV) is obtained from Equation (4) and is applied to quantify the difference between EDECO and the nine compared algorithms based on the Friedman rankings.

(20) $$CDV=q_{a}\times \sqrt{\frac{K\times \left(K+1\right)}{6N_{f}}}$$

where $q_{a}$ is equal to 3.164. K is set to 10 and $N_{f}$ is set to 29. CDV is equal to 2.3291 in this subsection. Based on the Friedman rankings and CDV, Figure 7 illustrates the magnitude of the differences between EDECO and competing algorithms, where there is no significant difference between the CDV-connected algorithms. As shown in Figure 7, EDECO significantly outperforms all algorithms on 10D, and shows a significant difference with the other compared algorithms other than ISGTOA/MRFO on 30D. For 50D and 100D, there is no significant difference between EDECO and ISGTOA, EMTLBO, MRFO, but it is significantly better than the remaining algorithms. Notably, the proposed EDECO is significantly better than the basic ECO in all dimensions, which shows the effectiveness of our work.

### 4.4. Analysis of the Results of Engineering Design Problems

To further check the performance of the EDECO algorithm in the face of constrained optimization problems, ten engineering design optimization problems were selected as challenges. These engineering problems are summarized in Table 7. EDECO, ECO, and other competitors are involved in the challenges. The maximum number of function evaluations is again 5000 times the problem dimension. The penalty function method was used to transform the constrained problems into unconstrained optimization problems. The results of 30 independent runs are recorded in Table 8.

**Figure 8:**
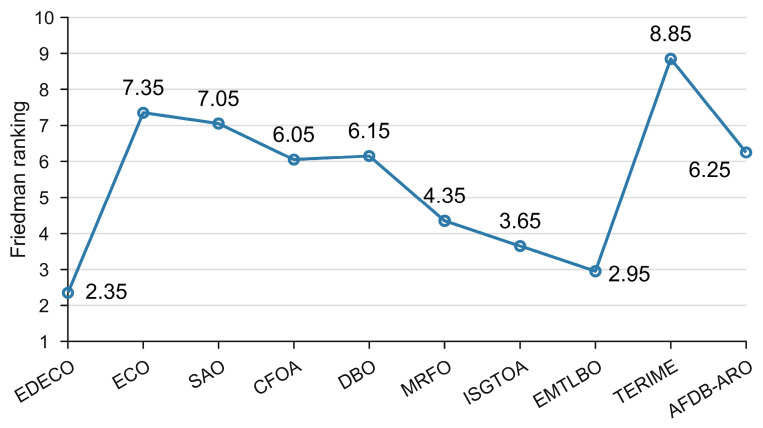
The Friedman ranking of EDECO and its competitors on constrained engineering optimization problems.

According to Table 8, we can observe that EDECO gives the best solutions to 5 problems (RW03, RW05, RW07, RW09, RW10) and ranks in the top 4 for the rest of these problems. basic ECO is ranked in the bottom 5 except for RW08. Similarly, this work created a Friedman test, as shown in Figure 8, which shows that EDECO achieves the best average ranking of 2.35 and that of ECO is 7.35. The Wilcoxon rank sum test also shows that EDECO achieves more “+” than “−”. In conclusion, EDECO successfully addresses a variety of real-world problems, demonstrating satisfactory performance. Its overall performance is highly competitive compared to other algorithms.

## 5. Conclusions

This work presents EDECO, a promising variant of ECO. The proposed EDECO combines an estimation of distribution algorithm through a hybrid search framework and incorporates a dynamic fitness distance balancing strategy to achieve a significant performance improvement. The excellent performance of EDECO is confirmed by a thorough comparison with advanced basic and improved algorithms. Experimental results on the CEC 2017 benchmarking functions show that EDECO outperforms SAO, CFOA, DBO, MRFO, ISGTOA, EMTLBO, TERIME, AFDB-ARO, and that there is a significant difference between it and the EDECO variants incorporating a single improvement technique. These results fully demonstrate that EDECO is a competitive metaheuristic variant that can efficiently face complex optimization problems with excellent convergence and robustness.

Although EDECO demonstrates promising optimization capabilities, as optimization problems continue to grow in complexity, there are still aspects of its design principles and methodologies that can be further enhanced. (1) Application to complex optimization problems: Future work could extend its application to areas such as mission planning (trajectory planning and target allocation), medical image segmentation, automated artistic design, and large-scale model architecture optimization. (2) Development of additional versions: Real-world problems are diverse and complex, necessitating the development of multi-objective versions of EDECO. Additionally, a binary version represents another promising research direction. (3) Further performance enhancement: While the proposed EDECO exhibits excellent overall performance, its suboptimal performance on certain functions motivates further improvements. We plan to refine the existing framework and explore integrating EDECO with other artificial intelligence techniques, such as reinforcement learning and deep learning. (4) Generalization of hybrid search framework: The hybrid search framework proposed in this work have proven effective in enhancing ECO’s performance. However, exploring their applicability to other metaheuristic algorithms may yield deeper insights and advancements.

## Figures and Tables

**Table 1:** Detailed description of CEC2017 test functions.

Test Suite	Type	No.	F_min_
CEC2017	Unimodal functions	F1–F3	100–300
	Multimodal functions	F4–F10	400–1000
	Hybrid functions	F11–F20	1100–2000
	Composition functions	F21–F30	2100–3000
Search range [−100, 100]			

**Table 2:** Parameter settings for ten algorithms.

Algorithm	Parameter Settings
EDECO	$H=0.5,G_{1}=0.2,G_{2}=0.1,\alpha =10,\beta =0.4$
ECO	$H=0.5,G_{1}=0.2,G_{2}=0.1$
SAO	$k=1,N_{1}=0.5N$
CFOA	p∈(3,4)
DBO	$P=0.2,r_{1}=0.9,\alpha =0.3,\beta =0.1$
MRFO	S=2
ISGTOA	Ar=2N
EMTLBO	$p_{ini}=0,p_{\min}=0.6,r_{1}=0.8$
TERIME	$w_{\max}=0.3,w_{\min}=0.0625$
AFDB-ARO	a=2,w=0.5

**Table 3:** The rankings of EDECO and its variants in CEC 2017 test suite according to the Friedman test (a = 0.05).

Test Suite	Dimension	ECO	EECO	DECO	EDECO	*p*-Value
CEC 2017	10	3.90	2.48	2.34	1.28	4.75E−13
	30	3.83	2.38	2.66	1.14	9.89E−14
	50	4.00	2.21	2.72	1.07	1.25E−16
	100	3.83	2.14	2.76	1.28	5.36E−13
Mean ranking		3.89	2.30	2.62	1.19	NaN

**Table 4:** The results of Wilcoxon rank sum test between EDECO and its variants (a = 0.05).

vs. ECO +/=/−	CEC-2017 Test Suite			
	10D	30D	50D	100D
EECO	22/6/1	19/10/0	20/9/0	21/8/0
DECO	21/7/1	15/12/2	17/12/0	19/10/0
EDECO	28/1/0	27/2/0	27/2/0	26/3/0

**Table 5:** The results of Wilcoxon rank sum test between EDECO and its competitors (a = 0.05).

EDECO vs. +/=/−	CEC-2017 Test Suite				Total
	10D	30D	50D	100D	
ECO	27/2/0	29/0/0	26/3/0	26/3/0	108/8/0
SAO	28/1/0	29/0/0	27/1/1	29/0/0	113/2/1
CFOA	22/6/1	28/1/0	27/2/0	29/0/0	106/9/1
DBO	25/4/0	27/2/0	27/2/0	28/0/1	107/8/1
MRFO	22/6/1	19/7/3	21/6/2	23/4/2	85/23/8
ISGTOA	22/6/1	13/12/4	10/9/10	12/9/8	57/36/23
EMTLBO	25/3/1	20/4/5	18/7/4	17/9/3	80/23/13
TERIME	24/5/0	26/3/0	27/2/0	28/1/0	105/11/0
AFDB-ARO	23/4/2	27/1/1	25/1/3	21/2/6	96/8/12

**Table 6:** The results of Friedman test between EDECO and its competitors.

Algorithm	CEC-2017 Test Suite					
	10D	30D	50D	100D	Mean Ranking	Overall Ranking
EDECO	1.8276	1.9655	2.2069	2.0690	2.0172	1
ECO	6.4483	8.6897	5.4138	5.1724	6.4310	7
SAO	8.3103	8.2069	8.7241	8.7931	8.5086	10
CFOA	5.1379	6.3793	7.0345	8.2759	6.7069	8
DBO	7.8276	6.7586	7.7931	7.7586	7.5345	9
MRFO	4.6207	3.5172	4.0000	4.2069	4.0862	3
ISGTOA	4.2414	2.4138	2.9655	3.3793	3.2500	2
EMTLBO	5.2759	4.4828	4.2414	4.1034	4.5259	4
TERIME	6.1724	6.2414	6.5172	6.7241	6.4138	6
AFDB-ARO	5.1379	6.3448	6.1034	4.5172	5.5259	5
*p*-value	5.00E−17	3.11E−28	2.81E−23	6.25E−27	N/A	N/A

**Table 7:** Ten real-world constrained engineering optimization problems.

Problem	Name	D
RW01	Tension/compression spring design problem	3
RW02	Pressure vessel design problem	4
RW03	Three-bar truss design problem	2
RW04	Welded beam design problem	4
RW05	Speed reducer design problem	7
RW06	Gear train design problem	4
RW07	Rolling element bearing design	10
RW08	Cantilever beam design problem	5
RW09	Multiple disk clutch brake design problem	5
RW10	Step-cone pulley problem	5

**Table 8:** Results of ten real-world constrained optimization problems obtained by EDECO and its competitors.

No.	Index	EDECO	ECO	SAO	CFOA	DBO	MRFO	ISGTOA	EMTLBO	TERIME	AFDB-ARO
RW1	Best	1.2669E−02	1.2685E−02	1.2738E−02	1.2674E−02	1.2719E−02	1.2758E−02	1.2686E−02	1.2666E−02	1.2791E−02	1.2820E−02
	Mean	1.2864E−02	1.3817E−02	1.2890E−02	1.2727E−02	1.4052E−02	1.3242E−02	1.2834E−02	1.2728E−02	1.5126E−02	1.3139E−02
	Std	7.5060E−04	1.4828E−03	1.7802E−04	1.8317E−05	1.7973E−03	3.6082E−04	9.2758E−05	7.9627E−05	2.4163E−03	1.9551E−04
	Rank	4	8	5	1	9	7	3	2	10	6
RW2	Best	5.8704E+03	5.9533E+03	5.8937E+03	5.8718E+03	5.8701E+03	5.8961E+03	5.9027E+03	5.8710E+03	6.1334E+03	5.9138E+03
	Mean	6.0663E+03	6.5032E+03	6.3398E+03	6.4378E+03	6.5134E+03	6.2741E+03	6.1319E+03	5.9778E+03	8.7195E+03	6.0595E+03
	Std	3.3002E+02	4.6657E+02	5.5444E+02	7.6231E+02	5.5236E+02	2.4423E+02	2.0576E+02	1.3536E+02	2.0960E+03	9.6526E+01
	Rank	3	8	6	7	9	5	4	1	10	2
RW3	Best	2.6389E+02	2.6389E+02	2.6390E+02	2.6389E+02	2.6389E+02	2.6389E+02	2.6389E+02	2.6389E+02	2.6389E+02	2.6389E+02
	Mean	2.6389E+02	2.6409E+02	2.6393E+02	2.6389E+02	2.6389E+02	2.6389E+02	2.6389E+02	2.6389E+02	2.6402E+02	2.6393E+02
	Std	4.4858E−10	4.1803E−01	2.5896E−02	7.7140E−03	2.1837E−03	1.3741E−03	2.4102E−03	3.5728E−04	1.4606E−01	2.4251E−02
	Rank	1	10	8	6	5	3	4	2	9	7
RW4	Best	1.6929E+00	1.7159E+00	1.7040E+00	1.7031E+00	1.6928E+00	1.6986E+00	1.7002E+00	1.6939E+00	1.7469E+00	1.7473E+00
	Mean	1.7078E+00	1.8940E+00	1.7334E+00	1.8028E+00	1.7246E+00	1.7098E+00	1.7125E+00	1.7018E+00	1.8904E+00	1.8253E+00
	Std	2.0634E−02	1.6516E−01	1.6138E−02	8.1996E−02	3.8621E−02	7.7329E−03	6.8060E−03	8.4140E−03	9.6649E−02	5.3845E−02
	Rank	2.00E+00	1.00E+01	6.00E+00	7.00E+00	5.00E+00	3.00E+00	4.00E+00	1.00E+00	9.00E+00	8.00E+00
RW5	Best	2.9936E+03	2.9936E+03	2.9953E+03	2.9937E+03	2.9936E+03	2.9951E+03	2.9936E+03	2.9936E+03	2.9943E+03	2.9942E+03
	Mean	2.9936E+03	2.9994E+03	3.0045E+03	3.0031E+03	2.9959E+03	2.9982E+03	2.9936E+03	2.9937E+03	3.0134E+03	2.9956E+03
	Std	1.0406E−04	6.3475E+00	5.2540E+00	7.7544E+00	4.6170E+00	1.8148E+00	1.4899E−04	2.4555E−02	1.6217E+01	1.3795E+00
	Rank	1	7	9	8	5	6	2	3	10	4
RW6	Best	2.7009E−12	2.7009E−12	2.3078E−11	2.7009E−12	2.3078E−11	2.7009E−12	2.7009E−12	2.7009E−12	2.7009E−12	2.7009E−12
	Mean	7.7801E−11	1.8517E−09	2.6352E−09	3.8504E−10	2.4014E−09	6.4834E−11	2.7514E−10	7.7968E−10	2.0626E−09	3.6345E−10
	Std	1.6781E−10	4.8789E−09	5.3234E−09	4.4710E−10	4.8591E−09	2.4769E−10	4.0326E−10	1.0022E−09	1.7757E−09	4.3973E−10
	Rank	2	7	10	5	9	1	3	6	8	4
RW7	Best	−2.4358E+05	−2.4358E+05	−2.4358E+05	−2.4358E+05	−2.4358E+05	−2.4319E+05	−2.4358E+05	−2.4358E+05	−2.4358E+05	−2.4334E+05
	Mean	−2.4358E+05	−2.4358E+05	−2.4358E+05	−2.4358E+05	−2.4358E+05	−2.4216E+05	−2.4358E+05	−2.4358E+05	−2.3989E+05	−2.4000E+05
	Std	6.7933E−11	5.2908E−02	3.1848E+00	2.4655E−02	1.6469E−05	7.1829E+02	7.1494E−11	1.1181E−10	3.8019E+03	2.3898E+03
	Rank	1	5	7	6	4	8	2	3	10	9
RW8	Best	1.3400E+00	1.3405E+00	1.3417E+00	1.3402E+00	1.3400E+00	1.3400E+00	1.3408E+00	1.3405E+00	1.3726E+00	1.3437E+00
	Mean	1.3404E+00	1.3831E+00	1.3455E+00	1.4912E+00	1.3403E+00	1.3404E+00	1.3421E+00	1.3416E+00	1.5795E+00	1.3497E+00
	Std	7.1854E−04	4.2430E−02	2.4454E−03	1.9531E−01	2.1076E−04	2.5067E−04	8.1659E−04	7.9139E−04	1.4039E−01	3.8672E−03
	Rank	3	8	6	9	1	2	5	4	10	7
RW9	Best	3.9247E+08	3.9247E+08	3.9247E+08	3.9247E+08	3.9247E+08	3.9247E+08	3.9247E+08	3.9247E+08	3.9247E+08	3.9247E+08
	Mean	3.9247E+08	3.9247E+08	3.9247E+08	3.9247E+08	3.9247E+08	3.9247E+08	3.9247E+08	3.9247E+08	3.9247E+08	3.9247E+08
	Std	1.8187E−07	1.8187E−07	1.8187E−07	1.8187E−07	1.8187E−07	1.8187E−07	1.8187E−07	1.8187E−07	1.8187E−07	1.8187E−07
	Rank	1	1	1	1	1	1	1	1	1	1
RW10	Best	1.6086E+01	1.6115E+01	1.8052E+01	1.6090E+01	1.6453E+01	1.6137E+01	1.6195E+01	1.6090E+01	1.6596E+01	1.8704E+01
	Mean	1.6090E+01	1.6634E+01	2.1916E+01	1.6714E+01	2.3561E+01	1.6481E+01	1.6625E+01	1.6180E+01	1.7934E+01	2.6097E+01
	Std	8.3474E−03	3.4454E−01	3.1844E+00	4.3513E−01	2.2653E+01	2.2656E−01	2.6490E−01	1.8328E−01	1.6910E+00	7.8801E+00
	Rank	1	5	8	6	9	3	4	2	7	10
Friedman ranking		2.35	7.35	7.05	6.05	6.15	4.35	3.65	2.95	8.85	6.25
Wilconxon rank sum results			9/1/0	9/1/0	8/2/0	7/3/0	7/1/2	8/1/1	6/4/0	9/1/0	8/1/1

## Data Availability

Data will be made available on request.

## Appendix A

**Table A1:** Results of CEC2017 10D functions obtained by EDECO and its competitors.

Function	Index	EDECO	ECO	SAO	CFOA	DBO	MRFO	ISGTOA	EMTLBO	TERIME	AFDB-ARO
F1	Best	1.0000E+02	1.6664E+02	1.9038E+07	2.7621E+05	3.2279E+02	6.0470E+02	1.1085E+02	2.4813E+02	1.5648E+06	5.8734E+03
	Mean	4.2957E+02	1.0700E+04	5.3372E+07	1.4643E+07	4.4217E+06	2.6891E+03	1.9579E+03	2.2673E+03	1.0659E+07	2.0313E+05
	Std	6.2647E+02	1.1597E+04	2.6754E+07	1.5378E+07	1.6834E+07	2.4956E+03	2.7804E+03	2.3118E+03	6.5850E+06	1.6624E+05
F3	Best	3.0000E+02	3.0007E+02	4.6260E+02	3.0394E+02	3.0868E+02	3.0011E+02	4.1405E+02	3.0078E+02	3.4832E+02	1.7321E+03
	Mean	3.0000E+02	3.2631E+02	6.5598E+02	7.6556E+02	8.5396E+02	3.0168E+02	6.8141E+02	4.3992E+02	5.8486E+02	4.5160E+03
	Std	8.6380E−04	7.5387E+01	1.2294E+02	5.0501E+02	6.2468E+02	1.1112E+00	2.0294E+02	3.5523E+02	1.7054E+02	1.4564E+03
F4	Best	4.0083E+02	4.0001E+02	4.0796E+02	4.0163E+02	4.0000E+02	4.0010E+02	4.0433E+02	4.0157E+02	4.0391E+02	4.0354E+02
	Mean	4.0302E+02	4.0668E+02	4.0987E+02	4.0625E+02	4.1696E+02	4.0522E+02	4.0743E+02	4.0450E+02	4.0964E+02	4.1974E+02
	Std	1.1668E+00	1.0929E+01	7.7098E−01	2.0550E+00	2.4834E+01	1.2324E+01	9.2394E+00	1.4797E+00	1.2395E+01	1.2834E+01
F5	Best	5.0205E+02	5.0897E+02	5.2317E+02	5.0724E+02	5.0982E+02	5.0601E+02	5.0504E+02	5.2007E+02	5.0643E+02	5.1335E+02
	Mean	5.0886E+02	5.2259E+02	5.3303E+02	5.1714E+02	5.2661E+02	5.1760E+02	5.1545E+02	5.2994E+02	5.2007E+02	5.2326E+02
	Std	4.2109E+00	8.1581E+00	4.5427E+00	5.7959E+00	8.5577E+00	8.3764E+00	7.0016E+00	4.4260E+00	7.2650E+00	5.6912E+00
F6	Best	6.0000E+02	6.0012E+02	6.0272E+02	6.0362E+02	6.0000E+02	6.0003E+02	6.0001E+02	6.0037E+02	6.0167E+02	6.0005E+02
	Mean	6.0002E+02	6.0601E+02	6.0485E+02	6.0793E+02	6.0094E+02	6.0007E+02	6.0001E+02	6.0057E+02	6.0375E+02	6.0085E+02
	Std	1.4928E−02	6.2573E+00	1.2954E+00	2.8036E+00	1.9655E+00	5.3539E−02	6.5965E−03	1.0889E−01	1.6294E+00	8.0903E−01
F7	Best	7.1297E+02	7.1539E+02	7.4139E+02	7.1608E+02	7.1373E+02	7.1541E+02	7.1794E+02	7.3063E+02	7.2433E+02	7.2238E+02
	Mean	7.1959E+02	7.3383E+02	7.5167E+02	7.2427E+02	7.3068E+02	7.3133E+02	7.3312E+02	7.3967E+02	7.4657E+02	7.3581E+02
	Std	3.7326E+00	1.0140E+01	6.3675E+00	4.7256E+00	1.1252E+01	1.0544E+01	8.0637E+00	4.1249E+00	9.8485E+00	7.0266E+00
F8	Best	8.0207E+02	8.0598E+02	8.1490E+02	8.0540E+02	8.0796E+02	8.0597E+02	8.0485E+02	8.1366E+02	8.1103E+02	8.0970E+02
	Mean	8.1035E+02	8.1797E+02	8.2523E+02	8.1346E+02	8.2270E+02	8.1615E+02	8.1618E+02	8.3007E+02	8.2427E+02	8.2193E+02
	Std	4.5733E+00	7.5822E+00	5.6420E+00	5.3749E+00	1.0380E+01	5.9493E+00	7.2464E+00	5.0655E+00	8.5204E+00	6.1574E+00
F9	Best	9.0000E+02	9.0011E+02	9.0261E+02	9.0200E+02	9.0000E+02	9.0000E+02	9.0000E+02	9.0002E+02	9.0114E+02	9.1127E+02
	Mean	9.0002E+02	9.4177E+02	9.0655E+02	9.2348E+02	9.0076E+02	9.0002E+02	9.0000E+02	9.0006E+02	9.1843E+02	9.2992E+02
	Std	8.4016E−02	7.7080E+01	2.6248E+00	1.4525E+01	2.6067E+00	3.5700E−02	1.2697E−03	3.6407E−02	2.5649E+01	1.2864E+01
F10	Best	1.1763E+03	1.3355E+03	1.2980E+03	1.2881E+03	1.1949E+03	1.1263E+03	1.4615E+03	1.5430E+03	1.0808E+03	1.3045E+03
	Mean	1.5839E+03	1.6756E+03	1.8609E+03	1.6783E+03	1.7094E+03	1.6669E+03	1.8856E+03	2.0739E+03	1.6977E+03	1.6604E+03
	Std	2.3352E+02	2.0139E+02	2.6603E+02	2.0948E+02	2.8601E+02	2.9769E+02	2.3528E+02	2.1640E+02	2.6144E+02	1.2447E+02
F11	Best	1.1005E+03	1.1099E+03	1.1219E+03	1.1110E+03	1.1020E+03	1.1034E+03	1.1038E+03	1.1037E+03	1.1028E+03	1.1162E+03
	Mean	1.1049E+03	1.1463E+03	1.1327E+03	1.1362E+03	1.1477E+03	1.1090E+03	1.1089E+03	1.1102E+03	1.1274E+03	1.1276E+03
	Std	2.0791E+00	4.7048E+01	6.4853E+00	1.8116E+01	6.7728E+01	3.2771E+00	3.4521E+00	3.2839E+00	3.6658E+01	7.8775E+00
F12	Best	1.2273E+03	3.2310E+03	2.6652E+05	5.6445E+03	2.6847E+03	3.3395E+03	6.2037E+03	1.7274E+03	8.0496E+04	1.5557E+04
	Mean	1.9576E+03	8.3363E+04	1.5718E+06	1.1211E+05	9.3190E+05	2.1834E+04	3.3965E+04	1.6690E+04	2.7915E+06	9.0221E+04
	Std	7.7352E+02	2.7194E+05	1.8713E+06	1.5945E+05	2.3340E+06	1.7449E+04	2.2321E+04	1.6265E+04	2.6210E+06	9.3890E+04
F13	Best	1.3059E+03	1.5457E+03	2.8950E+03	1.9407E+03	1.3609E+03	1.3623E+03	1.5193E+03	1.3172E+03	1.5933E+03	1.4298E+03
	Mean	1.3478E+03	2.3046E+03	1.1363E+04	3.0314E+03	1.2106E+04	2.5061E+03	2.0692E+03	1.6346E+03	1.6372E+04	1.6666E+03
	Std	4.8201E+01	3.5032E+02	6.9110E+03	7.3297E+02	1.2162E+04	1.6506E+03	4.1559E+02	7.9352E+02	1.1418E+04	1.5366E+02
F14	Best	1.4008E+03	1.4290E+03	1.4760E+03	1.4389E+03	1.4343E+03	1.4394E+03	1.4346E+03	1.4257E+03	1.4221E+03	1.4138E+03
	Mean	1.4168E+03	1.4769E+03	1.5483E+03	1.4680E+03	1.5093E+03	1.4845E+03	1.4559E+03	1.4709E+03	1.4706E+03	1.4303E+03
	Std	1.0433E+01	2.8275E+01	5.2516E+01	1.6764E+01	5.1298E+01	2.9414E+01	1.2335E+01	1.1109E+02	5.9134E+01	5.7099E+00
F15	Best	1.5006E+03	1.5386E+03	1.6127E+03	1.5468E+03	1.5133E+03	1.5582E+03	1.5365E+03	1.5067E+03	1.5251E+03	1.5252E+03
	Mean	1.5080E+03	1.6551E+03	1.8152E+03	1.6344E+03	1.9052E+03	1.6985E+03	1.5995E+03	1.6425E+03	2.1907E+03	1.5583E+03
	Std	9.6205E+00	8.6561E+01	2.2347E+02	5.7593E+01	4.0463E+02	1.1731E+02	3.9219E+01	4.3228E+02	8.1787E+02	1.7649E+01
F16	Best	1.6015E+03	1.6020E+03	1.6152E+03	1.6025E+03	1.6020E+03	1.6017E+03	1.6031E+03	1.6031E+03	1.6036E+03	1.6033E+03
	Mean	1.6138E+03	1.6832E+03	1.6642E+03	1.6681E+03	1.7351E+03	1.6792E+03	1.6259E+03	1.6551E+03	1.6702E+03	1.6757E+03
	Std	2.0573E+01	8.5165E+01	5.4847E+01	6.6510E+01	1.1525E+02	7.9501E+01	2.9800E+01	4.2808E+01	7.5269E+01	6.4833E+01
F17	Best	1.7175E+03	1.7231E+03	1.7334E+03	1.7240E+03	1.7216E+03	1.7068E+03	1.7304E+03	1.7076E+03	1.7087E+03	1.7189E+03
	Mean	1.7418E+03	1.7529E+03	1.7592E+03	1.7436E+03	1.7586E+03	1.7386E+03	1.7527E+03	1.7442E+03	1.7393E+03	1.7347E+03
	Std	1.1004E+01	1.5976E+01	1.0842E+01	1.1883E+01	3.1297E+01	1.5425E+01	1.1949E+01	3.6363E+01	1.1835E+01	9.1603E+00
F18	Best	1.8071E+03	1.8742E+03	8.4281E+03	2.1484E+03	2.1990E+03	1.9932E+03	2.1726E+03	1.8453E+03	4.2416E+03	1.9191E+03
	Mean	1.8384E+03	5.1728E+03	3.4097E+04	4.7255E+03	1.6523E+04	7.7965E+03	4.3282E+03	6.0126E+03	2.4234E+04	2.5527E+03
	Std	2.7479E+01	4.7343E+03	1.3868E+04	2.2810E+03	1.4014E+04	5.7300E+03	2.8115E+03	6.5034E+03	1.4291E+04	8.8030E+02
F19	Best	1.9008E+03	1.9091E+03	1.9390E+03	1.9200E+03	1.9078E+03	1.9170E+03	1.9111E+03	1.9058E+03	1.9123E+03	1.9113E+03
	Mean	1.9041E+03	1.9516E+03	2.8731E+03	1.9632E+03	3.5221E+03	2.0876E+03	1.9438E+03	2.5114E+03	3.1538E+03	1.9264E+03
	Std	1.3937E+00	3.6330E+01	1.7041E+03	3.8064E+01	5.6984E+03	1.8673E+02	2.7025E+01	2.3483E+03	2.4763E+03	8.8024E+00
F20	Best	2.0225E+03	2.0226E+03	2.0468E+03	2.0268E+03	2.0003E+03	2.0040E+03	2.0121E+03	2.0488E+03	2.0243E+03	2.0026E+03
	Mean	2.0427E+03	2.0675E+03	2.0599E+03	2.0472E+03	2.0393E+03	2.0236E+03	2.0371E+03	2.0617E+03	2.0458E+03	2.0229E+03
	Std	1.4418E+01	2.2512E+01	6.7939E+00	1.3437E+01	3.0802E+01	1.6200E+01	1.2079E+01	8.4457E+00	1.8892E+01	9.2740E+00
F21	Best	2.2000E+03	2.2004E+03	2.2026E+03	2.2031E+03	2.1000E+03	2.1006E+03	2.2006E+03	2.2033E+03	2.2028E+03	2.2025E+03
	Mean	2.2009E+03	2.2122E+03	2.2185E+03	2.2070E+03	2.2058E+03	2.2095E+03	2.2486E+03	2.2095E+03	2.2044E+03	2.2182E+03
	Std	1.2701E+00	3.4297E+01	3.8974E+01	3.0528E+00	2.9369E+01	4.0516E+01	5.7567E+01	2.0731E+01	1.0412E+00	1.8449E+01
F22	Best	2.2000E+03	2.2001E+03	2.2452E+03	2.2214E+03	2.2258E+03	2.2002E+03	2.2000E+03	2.2096E+03	2.2329E+03	2.2443E+03
	Mean	2.2484E+03	2.2592E+03	2.3049E+03	2.3043E+03	2.2988E+03	2.2900E+03	2.2866E+03	2.2898E+03	2.2849E+03	2.2786E+03
	Std	3.9288E+01	4.0937E+01	2.4568E+01	2.1868E+01	2.9958E+01	3.1154E+01	3.3905E+01	3.3679E+01	3.3443E+01	1.9401E+01
F23	Best	2.6039E+03	2.6104E+03	2.6247E+03	2.3057E+03	2.6112E+03	2.6081E+03	2.6073E+03	2.6148E+03	2.6116E+03	2.3054E+03
	Mean	2.6135E+03	2.6256E+03	2.6333E+03	2.5791E+03	2.6304E+03	2.6179E+03	2.6164E+03	2.6288E+03	2.6244E+03	2.6242E+03
	Std	5.0863E+00	1.1549E+01	4.9813E+00	1.0848E+02	1.0260E+01	6.3946E+00	6.6986E+00	5.2466E+00	9.3523E+00	6.0964E+01
F24	Best	2.5000E+03	2.5002E+03	2.5091E+03	2.4619E+03	2.5000E+03	2.5000E+03	2.5004E+03	2.5492E+03	2.4548E+03	2.4878E+03
	Mean	2.6513E+03	2.6684E+03	2.6843E+03	2.5621E+03	2.6660E+03	2.6584E+03	2.6888E+03	2.7003E+03	2.5866E+03	2.5564E+03
	Std	1.1707E+02	1.1922E+02	1.1885E+02	4.8869E+01	1.1388E+02	1.1772E+02	1.0547E+02	9.2779E+01	1.1624E+02	5.0899E+01
F25	Best	2.8977E+03	2.8978E+03	2.8984E+03	2.8985E+03	2.8981E+03	2.8978E+03	2.8979E+03	2.8980E+03	2.8993E+03	2.6677E+03
	Mean	2.9212E+03	2.9242E+03	2.9361E+03	2.9042E+03	2.9350E+03	2.9217E+03	2.9219E+03	2.9146E+03	2.9315E+03	2.8863E+03
	Std	2.3397E+01	2.3309E+01	2.0725E+01	6.8861E+00	2.7293E+01	2.3395E+01	2.3470E+01	2.1658E+01	2.4599E+01	7.3027E+01
F26	Best	2.9000E+03	2.9001E+03	2.9251E+03	2.9143E+03	2.9000E+03	2.9000E+03	2.9000E+03	2.9002E+03	2.8986E+03	2.7746E+03
	Mean	2.9000E+03	2.9496E+03	2.9508E+03	2.9626E+03	3.0151E+03	2.9063E+03	2.9125E+03	2.9012E+03	2.9334E+03	2.9420E+03
	Std	2.9357E−03	8.7581E+01	1.5971E+01	4.0287E+01	4.3364E+01	2.2587E+01	2.4017E+01	6.0566E−01	3.1184E+01	7.3499E+01
F27	Best	3.0892E+03	3.0894E+03	3.0903E+03	3.0899E+03	3.0904E+03	3.0901E+03	3.0890E+03	3.0904E+03	3.0908E+03	3.1064E+03
	Mean	3.0914E+03	3.0938E+03	3.0953E+03	3.0955E+03	3.0964E+03	3.0981E+03	3.0902E+03	3.0914E+03	3.0965E+03	3.1135E+03
	Std	2.5005E+00	3.0748E+00	1.5795E+00	3.0515E+00	3.4404E+00	4.8215E+00	9.8906E−01	6.4803E−01	3.1208E+00	4.2835E+00
F28	Best	3.1000E+03	3.1003E+03	3.1614E+03	3.1208E+03	3.1691E+03	3.1001E+03	3.1003E+03	3.1050E+03	3.1208E+03	3.0258E+03
	Mean	3.2417E+03	3.3182E+03	3.2439E+03	3.1868E+03	3.2769E+03	3.1762E+03	3.2228E+03	3.1616E+03	3.1841E+03	3.2103E+03
	Std	1.2722E+02	1.2387E+02	1.0220E+02	2.8216E+01	1.0483E+02	1.1853E+02	1.1112E+02	8.1973E+01	2.2536E+01	7.1913E+01
F29	Best	3.1382E+03	3.1379E+03	3.1725E+03	3.1433E+03	3.1581E+03	3.1463E+03	3.1486E+03	3.1487E+03	3.1513E+03	3.1526E+03
	Mean	3.1613E+03	3.2046E+03	3.1970E+03	3.1831E+03	3.2039E+03	3.1890E+03	3.1867E+03	3.1937E+03	3.1825E+03	3.2028E+03
	Std	1.6949E+01	5.4528E+01	1.6699E+01	2.3472E+01	4.8482E+01	2.9891E+01	1.8095E+01	3.2183E+01	1.9909E+01	1.9113E+01
F30	Best	3.5310E+03	4.0582E+03	1.5663E+04	4.0153E+03	3.7068E+03	4.2384E+03	6.5611E+03	3.7827E+03	7.7210E+03	1.6514E+04
	Mean	1.7054E+04	1.5101E+05	3.8045E+05	4.7171E+04	5.6042E+05	3.0943E+05	1.4982E+05	1.9457E+05	1.2525E+05	3.1662E+05
	Std	5.4232E+04	2.6223E+05	4.6557E+05	6.5635E+04	4.7247E+05	5.9605E+05	2.0978E+05	3.3016E+05	1.8985E+05	2.8525E+05

**Table A2:** Results of CEC2017 30D functions obtained by EDECO and its competitors.

Function	Index	EDECO	ECO	SAO	CFOA	DBO	MRFO	ISGTOA	EMTLBO	TERIME	AFDB-ARO
F1	Best	3.5853E+03	1.8798E+08	1.5334E+09	1.4219E+09	1.1622E+05	1.4842E+05	4.5167E+02	4.4013E+04	1.9993E+08	5.3758E+04
	Mean	4.9269E+04	6.0544E+09	2.2325E+09	2.5321E+09	6.6359E+07	3.1884E+05	3.9321E+03	1.4834E+05	5.7717E+08	2.4551E+05
	Std	4.7262E+04	6.0700E+09	3.8007E+08	6.7906E+08	4.2155E+07	1.0483E+05	3.5069E+03	1.2583E+05	2.2459E+08	2.5486E+05
F3	Best	4.7261E+02	3.9840E+03	2.1904E+04	1.1979E+04	3.1055E+04	3.2941E+03	1.6652E+04	9.3023E+03	1.7843E+04	5.3358E+04
	Mean	1.4839E+03	2.0230E+04	3.8791E+04	2.5105E+04	6.0561E+04	7.6418E+03	2.9248E+04	2.3871E+04	3.0373E+04	6.8846E+04
	Std	1.1725E+03	1.3013E+04	6.7160E+03	8.1251E+03	1.2430E+04	2.3586E+03	5.6130E+03	1.0428E+04	7.4690E+03	7.4843E+03
F4	Best	4.7390E+02	4.9456E+02	6.0635E+02	6.0970E+02	5.0414E+02	4.7704E+02	4.7324E+02	4.0530E+02	5.2611E+02	4.8858E+02
	Mean	5.0551E+02	1.0444E+03	6.6682E+02	8.0685E+02	5.5655E+02	5.1327E+02	5.0931E+02	4.9537E+02	5.8357E+02	5.8445E+02
	Std	1.9095E+01	6.6393E+02	3.5430E+01	1.1865E+02	4.5631E+01	2.3040E+01	2.3799E+01	2.7206E+01	4.1930E+01	5.5177E+01
F5	Best	5.4094E+02	6.0573E+02	6.9230E+02	6.3193E+02	5.9806E+02	5.6223E+02	5.4172E+02	6.6241E+02	5.9825E+02	6.1479E+02
	Mean	5.8542E+02	7.1637E+02	7.2215E+02	6.5977E+02	6.6218E+02	6.2209E+02	5.7493E+02	6.8169E+02	6.4807E+02	6.5841E+02
	Std	2.8339E+01	6.1073E+01	1.1111E+01	1.6024E+01	3.5511E+01	3.3567E+01	2.2021E+01	1.1742E+01	2.7103E+01	2.2357E+01
F6	Best	6.0106E+02	6.2603E+02	6.1583E+02	6.2499E+02	6.0190E+02	6.0108E+02	6.0015E+02	6.0131E+02	6.0795E+02	6.0028E+02
	Mean	6.0475E+02	6.4938E+02	6.2051E+02	6.3385E+02	6.1638E+02	6.0723E+02	6.0108E+02	6.0239E+02	6.1435E+02	6.0401E+02
	Std	3.9282E+00	1.0213E+01	2.7081E+00	4.7430E+00	9.1168E+00	6.2859E+00	8.9407E−01	6.3896E−01	3.5540E+00	1.8552E+00
F7	Best	7.6834E+02	9.4425E+02	9.6284E+02	8.7955E+02	7.8856E+02	8.0939E+02	7.9933E+02	8.9880E+02	8.8523E+02	8.2939E+02
	Mean	8.4154E+02	1.0388E+03	9.9582E+02	9.2468E+02	8.8444E+02	8.9657E+02	8.6537E+02	9.1698E+02	9.5985E+02	9.2549E+02
	Std	3.1560E+01	7.7198E+01	1.7530E+01	2.4418E+01	5.7465E+01	4.6704E+01	3.4335E+01	8.9337E+00	3.7372E+01	3.3806E+01
F8	Best	8.3509E+02	8.6123E+02	9.7549E+02	8.8974E+02	8.9655E+02	8.7124E+02	8.4981E+02	9.5806E+02	8.9688E+02	8.9611E+02
	Mean	8.7371E+02	9.7668E+02	1.0081E+03	9.3058E+02	9.6214E+02	9.1229E+02	8.9826E+02	9.8264E+02	9.5883E+02	9.4937E+02
	Std	2.2744E+01	5.3143E+01	1.4282E+01	1.7267E+01	4.1875E+01	2.9953E+01	4.1726E+01	1.0425E+01	3.2728E+01	2.9174E+01
F9	Best	9.0433E+02	3.2233E+03	1.5184E+03	2.0071E+03	1.8011E+03	9.7482E+02	9.0642E+02	9.0232E+02	1.6121E+03	1.4160E+03
	Mean	1.1556E+03	4.7235E+03	1.9180E+03	2.6645E+03	4.1434E+03	2.2691E+03	9.2449E+02	9.0617E+02	2.9295E+03	3.5874E+03
	Std	3.6346E+02	1.1065E+03	2.2971E+02	3.3270E+02	1.7601E+03	1.0866E+03	1.5994E+01	3.1675E+00	1.0727E+03	8.4869E+02
F10	Best	4.2899E+03	4.6992E+03	6.6048E+03	4.4764E+03	4.0126E+03	3.3620E+03	4.5943E+03	6.8829E+03	4.7262E+03	3.1511E+03
	Mean	5.4251E+03	6.8069E+03	7.8792E+03	5.7604E+03	5.2110E+03	4.6535E+03	7.1802E+03	7.9964E+03	5.3581E+03	4.4002E+03
	Std	4.8742E+02	1.2070E+03	4.3006E+02	4.8723E+02	1.0125E+03	8.3787E+02	9.6906E+02	3.1933E+02	3.8398E+02	4.9661E+02
F11	Best	1.1516E+03	1.3029E+03	1.4707E+03	1.4554E+03	1.2425E+03	1.1692E+03	1.2184E+03	1.1846E+03	1.2422E+03	1.3013E+03
	Mean	1.2058E+03	1.7200E+03	1.5712E+03	2.0863E+03	1.4768E+03	1.2524E+03	1.2883E+03	1.2435E+03	1.3030E+03	1.3891E+03
	Std	3.8199E+01	4.3220E+02	6.4426E+01	4.2008E+02	1.1410E+02	3.8805E+01	3.3773E+01	3.6881E+01	3.3278E+01	5.3945E+01
F12	Best	8.4870E+04	8.8398E+06	1.2822E+08	1.6513E+07	2.6367E+05	1.3412E+05	1.0270E+05	5.6330E+04	5.8909E+06	1.0669E+06
	Mean	1.1786E+06	2.3229E+08	1.9003E+08	4.3437E+07	2.0950E+07	9.1000E+05	6.3194E+05	4.6360E+05	2.9142E+07	3.7432E+06
	Std	1.1725E+06	2.7811E+08	4.6227E+07	2.1107E+07	2.2789E+07	6.1352E+05	5.5558E+05	5.2563E+05	1.9110E+07	1.8801E+06
F13	Best	4.2787E+03	2.9940E+04	1.1349E+07	1.9964E+04	9.0004E+03	1.7716E+03	1.9850E+03	3.1097E+03	2.6020E+05	2.8668E+04
	Mean	1.7099E+04	1.5274E+05	4.7321E+07	3.8128E+05	2.5876E+06	1.3795E+04	1.6967E+04	1.8384E+04	2.3864E+06	8.3025E+04
	Std	1.0988E+04	1.1931E+05	2.2350E+07	4.0397E+05	1.1126E+07	1.4773E+04	1.5633E+04	1.7343E+04	2.0286E+06	4.7870E+04
F14	Best	1.4692E+03	1.7132E+03	7.6594E+03	3.2079E+03	5.1374E+03	1.9696E+03	2.2725E+03	1.5551E+03	4.1630E+03	2.6925E+03
	Mean	1.5381E+03	1.0688E+04	5.7076E+04	1.3681E+04	7.0775E+04	8.9148E+03	5.6761E+03	1.0653E+04	5.5694E+04	1.6611E+04
	Std	4.0604E+01	2.2994E+04	3.2445E+04	9.6129E+03	7.2087E+04	1.0114E+04	3.6488E+03	1.4796E+04	5.1384E+04	2.1909E+04
F15	Best	1.7917E+03	8.0654E+03	1.8656E+05	5.0579E+03	5.6375E+03	1.6862E+03	1.9668E+03	1.8370E+03	4.9155E+04	3.5552E+03
	Mean	2.6605E+03	3.8155E+04	6.4030E+05	4.2980E+04	5.2732E+04	1.3594E+04	8.4133E+03	5.9693E+03	4.2753E+05	1.0271E+04
	Std	1.1536E+03	2.5747E+04	4.5952E+05	8.5200E+04	4.4799E+04	1.1359E+04	6.9659E+03	8.0073E+03	5.8961E+05	8.5133E+03
F16	Best	1.8764E+03	2.4560E+03	2.5964E+03	2.3083E+03	2.1511E+03	1.9740E+03	1.8135E+03	2.2495E+03	2.0119E+03	2.3131E+03
	Mean	2.3550E+03	3.2266E+03	3.0932E+03	2.6917E+03	2.8233E+03	2.4829E+03	2.5431E+03	2.9768E+03	2.6158E+03	2.8355E+03
	Std	2.0524E+02	4.2048E+02	2.3477E+02	2.3150E+02	2.5899E+02	2.6349E+02	3.5624E+02	3.3758E+02	2.6273E+02	2.1094E+02
F17	Best	1.7752E+03	1.8233E+03	1.9310E+03	1.8474E+03	1.9575E+03	1.7622E+03	1.8091E+03	1.8461E+03	1.8045E+03	1.8066E+03
	Mean	1.8665E+03	2.4374E+03	2.0905E+03	1.9948E+03	2.3040E+03	1.9995E+03	1.9514E+03	2.0727E+03	2.1190E+03	2.1496E+03
	Std	5.5747E+01	2.9224E+02	1.0785E+02	1.1028E+02	1.9838E+02	1.5034E+02	8.7658E+01	1.4285E+02	1.6418E+02	1.6868E+02
F18	Best	2.0216E+03	3.3520E+04	2.3171E+05	3.5239E+04	6.3540E+04	3.9240E+04	3.9583E+04	2.0502E+04	6.9762E+04	3.8799E+04
	Mean	4.4653E+03	1.4280E+05	1.0518E+06	1.7164E+05	1.0691E+06	2.3526E+05	1.4651E+05	1.8297E+05	1.0907E+06	2.4321E+05
	Std	4.1174E+03	1.2811E+05	6.6436E+05	9.0793E+04	1.6016E+06	2.1715E+05	9.5910E+04	2.1689E+05	1.0467E+06	1.4595E+05
F19	Best	1.9578E+03	7.3817E+03	8.2549E+05	4.3255E+03	2.3272E+03	1.9680E+03	2.0895E+03	2.1009E+03	4.5360E+04	3.0313E+03
	Mean	2.3392E+03	6.9921E+05	3.0986E+06	4.6557E+04	2.5519E+05	1.1806E+04	6.7214E+03	7.4914E+03	5.7122E+05	9.6765E+03
	Std	7.4841E+02	1.4884E+06	2.1506E+06	4.4514E+04	5.2710E+05	1.1027E+04	6.8501E+03	6.5254E+03	6.3471E+05	5.8900E+03
F20	Best	2.1398E+03	2.3796E+03	2.2267E+03	2.1611E+03	2.1074E+03	2.1354E+03	2.0708E+03	2.4872E+03	2.0876E+03	2.2057E+03
	Mean	2.2998E+03	2.7301E+03	2.3706E+03	2.3951E+03	2.4172E+03	2.3597E+03	2.3705E+03	2.6765E+03	2.3045E+03	2.4704E+03
	Std	8.1987E+01	1.6670E+02	9.9238E+01	1.0307E+02	1.6437E+02	1.5060E+02	1.5837E+02	9.3655E+01	1.4405E+02	1.2810E+02
F21	Best	2.3294E+03	2.3802E+03	2.4792E+03	2.2712E+03	2.4013E+03	2.3588E+03	2.3397E+03	2.4519E+03	2.4056E+03	2.4247E+03
	Mean	2.3717E+03	2.4880E+03	2.5030E+03	2.4282E+03	2.4694E+03	2.3944E+03	2.3921E+03	2.4753E+03	2.4423E+03	2.4742E+03
	Std	2.2960E+01	4.3173E+01	1.1959E+01	3.3935E+01	3.4156E+01	2.2091E+01	3.0322E+01	1.2087E+01	2.1659E+01	3.0114E+01
F22	Best	2.3011E+03	2.6138E+03	2.5181E+03	2.4980E+03	2.3331E+03	2.3043E+03	2.3000E+03	2.3114E+03	2.3592E+03	2.3753E+03
	Mean	2.5841E+03	6.9933E+03	2.6302E+03	2.7315E+03	3.5236E+03	2.3082E+03	2.3009E+03	2.3170E+03	3.2830E+03	3.7056E+03
	Std	1.0617E+03	2.3481E+03	4.7988E+01	1.2306E+02	1.8174E+03	3.2849E+00	2.5812E+00	7.7546E+00	1.7647E+03	7.5454E+02
F23	Best	2.6871E+03	2.7892E+03	2.8429E+03	2.7697E+03	2.7630E+03	2.7216E+03	2.6702E+03	2.7831E+03	2.7607E+03	2.7231E+03
	Mean	2.7302E+03	3.0693E+03	2.8767E+03	2.8174E+03	2.8258E+03	2.7707E+03	2.7270E+03	2.8307E+03	2.8108E+03	2.8776E+03
	Std	3.0587E+01	1.5218E+02	1.5796E+01	2.2455E+01	3.2286E+01	3.1139E+01	3.5226E+01	1.4477E+01	2.9073E+01	4.0662E+01
F24	Best	2.8477E+03	2.9981E+03	3.0135E+03	2.9160E+03	2.9314E+03	2.8796E+03	2.8518E+03	2.9819E+03	2.9438E+03	2.9449E+03
	Mean	2.8937E+03	3.2848E+03	3.0403E+03	2.9712E+03	3.0139E+03	2.9490E+03	2.8973E+03	2.9983E+03	3.0064E+03	3.0613E+03
	Std	2.3362E+01	2.6656E+02	1.2813E+01	2.3655E+01	4.1297E+01	4.7133E+01	2.9738E+01	9.8678E+00	3.0880E+01	4.3458E+01
F25	Best	2.8837E+03	2.9523E+03	2.9525E+03	3.0171E+03	2.8836E+03	2.8854E+03	2.8838E+03	2.8864E+03	2.9093E+03	2.8925E+03
	Mean	2.8930E+03	3.1020E+03	2.9840E+03	3.0752E+03	2.9278E+03	2.9080E+03	2.8973E+03	2.8981E+03	2.9688E+03	2.9471E+03
	Std	1.1365E+01	1.3885E+02	1.9704E+01	2.6449E+01	2.7584E+01	1.6994E+01	1.3843E+01	1.3010E+01	3.4831E+01	3.2337E+01
F26	Best	2.9026E+03	3.9479E+03	5.6661E+03	3.6904E+03	3.5683E+03	2.8151E+03	2.9005E+03	2.8971E+03	3.6141E+03	3.2338E+03
	Mean	4.2223E+03	6.6306E+03	5.8661E+03	4.5460E+03	5.3572E+03	4.4276E+03	4.2369E+03	5.0101E+03	5.2988E+03	4.8218E+03
	Std	6.7182E+02	1.3141E+03	1.2924E+02	7.1305E+02	7.6308E+02	1.1087E+03	4.2625E+02	8.5062E+02	4.5702E+02	6.6758E+02
F27	Best	3.1981E+03	3.2259E+03	3.2299E+03	3.2497E+03	3.2144E+03	3.2275E+03	3.1920E+03	3.1967E+03	3.2297E+03	3.2395E+03
	Mean	3.2287E+03	3.4130E+03	3.2472E+03	3.2838E+03	3.2463E+03	3.2622E+03	3.2231E+03	3.2183E+03	3.2468E+03	3.2861E+03
	Std	1.5952E+01	1.3752E+02	9.1073E+00	2.0281E+01	1.9538E+01	2.5217E+01	1.2978E+01	9.9354E+00	1.1614E+01	2.7234E+01
F28	Best	3.2036E+03	3.3439E+03	3.3620E+03	3.3355E+03	3.2783E+03	3.2054E+03	3.2057E+03	3.2044E+03	3.2564E+03	3.2517E+03
	Mean	3.2358E+03	3.8122E+03	3.4150E+03	3.4571E+03	3.3696E+03	3.2387E+03	3.2381E+03	3.2395E+03	3.3282E+03	3.3048E+03
	Std	2.0493E+01	3.5926E+02	2.8925E+01	5.2352E+01	5.8336E+01	1.9556E+01	2.2474E+01	2.6919E+01	3.4461E+01	4.4028E+01
F29	Best	3.4413E+03	4.1395E+03	3.6585E+03	3.6709E+03	3.4487E+03	3.4508E+03	3.4773E+03	3.5604E+03	3.4460E+03	3.9445E+03
	Mean	3.7831E+03	4.7846E+03	4.0276E+03	4.0191E+03	3.8829E+03	3.7543E+03	3.7063E+03	3.9275E+03	3.7678E+03	4.2511E+03
	Std	2.0069E+02	3.8145E+02	1.9800E+02	1.9051E+02	2.3622E+02	1.7281E+02	1.5409E+02	2.2096E+02	2.0310E+02	1.8549E+02
F30	Best	7.0601E+03	3.7739E+05	4.0037E+06	7.0087E+04	8.7955E+03	1.0040E+04	1.1231E+04	7.7881E+03	1.4603E+05	7.4578E+04
	Mean	2.6531E+04	7.7681E+06	1.1103E+07	9.3399E+05	7.1791E+05	3.2885E+04	2.5177E+04	2.1048E+04	1.1605E+06	1.3491E+05
	Std	1.2868E+04	8.4947E+06	3.3596E+06	7.0461E+05	1.5598E+06	2.4483E+04	1.3490E+04	1.0263E+04	8.1768E+05	5.5937E+04

**Table A3:** Results of CEC2017 50D functions obtained by EDECO and its competitors.

Function	Index	EDECO	ECO	SAO	CFOA	DBO	MRFO	ISGTOA	EMTLBO	TERIME	AFDB-ARO
F1	Best	1.4571E+06	5.6577E+07	6.9231E+09	9.5444E+09	3.2487E+08	5.1922E+06	4.0229E+04	7.6106E+05	1.9259E+09	2.2990E+06
	Mean	3.5528E+06	1.8317E+08	9.3995E+09	1.4547E+10	1.3196E+09	1.1478E+07	2.5893E+05	3.4394E+06	4.1220E+09	6.5651E+06
	Std	1.4347E+06	1.2870E+08	1.3249E+09	2.9557E+09	6.8850E+08	4.1979E+06	2.2062E+05	1.5875E+06	1.1922E+09	3.1512E+06
F3	Best	3.0061E+03	8.6664E+03	6.6131E+04	4.5934E+04	8.4925E+04	3.5865E+04	6.6214E+04	2.8709E+04	6.0095E+04	1.3451E+05
	Mean	1.1319E+04	2.6586E+04	1.0712E+05	7.1119E+04	1.5901E+05	6.7079E+04	8.5481E+04	5.9353E+04	1.0348E+05	1.6300E+05
	Std	4.4909E+03	1.0144E+04	1.4259E+04	1.2932E+04	2.5369E+04	1.5212E+04	8.7477E+03	1.8463E+04	1.6173E+04	1.1859E+04
F4	Best	4.9273E+02	4.9158E+02	1.1474E+03	1.3540E+03	6.1612E+02	5.1473E+02	5.0293E+02	4.7347E+02	7.7844E+02	5.7754E+02
	Mean	6.0056E+02	6.7618E+02	1.4520E+03	2.1145E+03	8.8836E+02	6.3204E+02	5.7501E+02	5.9728E+02	9.8306E+02	7.1280E+02
	Std	4.8819E+01	6.0625E+01	1.7027E+02	3.3981E+02	1.7182E+02	5.4772E+01	4.3377E+01	4.6562E+01	1.9798E+02	6.4158E+01
F5	Best	6.3297E+02	6.7207E+02	8.9488E+02	7.9229E+02	6.9434E+02	7.2676E+02	6.0359E+02	8.3962E+02	7.5360E+02	7.4068E+02
	Mean	7.0237E+02	7.7635E+02	9.4910E+02	8.4085E+02	8.5440E+02	7.8957E+02	6.7864E+02	8.7298E+02	8.2588E+02	8.1373E+02
	Std	4.3790E+01	4.7648E+01	2.2381E+01	2.7446E+01	6.4625E+01	3.4239E+01	6.0281E+01	1.4400E+01	3.9934E+01	4.0320E+01
F6	Best	6.0578E+02	6.3479E+02	6.2785E+02	6.3827E+02	6.1516E+02	6.1000E+02	6.0292E+02	6.0236E+02	6.1576E+02	6.0198E+02
	Mean	6.1694E+02	6.4996E+02	6.3288E+02	6.4797E+02	6.3491E+02	6.3005E+02	6.0658E+02	6.0417E+02	6.2450E+02	6.0570E+02
	Std	7.5902E+00	8.0826E+00	2.7969E+00	4.4560E+00	8.5352E+00	1.4432E+01	2.5376E+00	9.4614E−01	4.9206E+00	2.0383E+00
F7	Best	9.1220E+02	1.1344E+03	1.2276E+03	1.1674E+03	9.9536E+02	1.0107E+03	8.7259E+02	1.1104E+03	1.1241E+03	9.5697E+02
	Mean	1.0335E+03	1.2648E+03	1.3052E+03	1.2558E+03	1.1415E+03	1.1562E+03	1.0068E+03	1.1384E+03	1.2832E+03	1.1418E+03
	Std	8.8554E+01	9.4668E+01	3.0312E+01	4.4444E+01	1.1124E+02	9.5887E+01	7.4750E+01	1.6057E+01	6.4815E+01	6.5160E+01
F8	Best	9.3147E+02	1.0026E+03	1.2144E+03	1.0687E+03	1.0298E+03	9.7591E+02	8.8803E+02	1.1343E+03	1.0215E+03	1.0093E+03
	Mean	9.9433E+02	1.1059E+03	1.2508E+03	1.1468E+03	1.1652E+03	1.0917E+03	9.7380E+02	1.1756E+03	1.1331E+03	1.1115E+03
	Std	4.0889E+01	4.8825E+01	2.1391E+01	3.7597E+01	7.3935E+01	4.3284E+01	4.4825E+01	1.5611E+01	4.6594E+01	4.0203E+01
F9	Best	1.6079E+03	4.9280E+03	4.2648E+03	6.4620E+03	6.3920E+03	4.3773E+03	1.0310E+03	9.2793E+02	4.2940E+03	9.9808E+03
	Mean	3.0381E+03	1.1144E+04	6.1216E+03	1.0011E+04	1.5787E+04	1.1545E+04	1.6295E+03	1.0162E+03	8.7111E+03	1.4154E+04
	Std	1.1013E+03	3.0185E+03	9.3311E+02	1.7321E+03	5.5372E+03	4.3055E+03	6.0065E+02	7.9423E+01	2.6925E+03	2.1460E+03
F10	Best	7.3194E+03	7.0215E+03	1.2577E+04	9.1524E+03	6.4686E+03	6.1631E+03	1.1259E+04	1.3361E+04	7.7493E+03	6.0613E+03
	Mean	8.8318E+03	9.1607E+03	1.3564E+04	1.0522E+04	9.0607E+03	7.7242E+03	1.3501E+04	1.4194E+04	9.3570E+03	7.2025E+03
	Std	9.6066E+02	1.1235E+03	5.7587E+02	8.0518E+02	1.9612E+03	1.0677E+03	9.9227E+02	3.8259E+02	8.3628E+02	6.4934E+02
F11	Best	1.2156E+03	1.3403E+03	2.3599E+03	2.2379E+03	1.6858E+03	1.3214E+03	1.5665E+03	1.3444E+03	1.4841E+03	1.5422E+03
	Mean	1.3152E+03	1.4850E+03	3.5760E+03	6.1667E+03	2.1549E+03	1.4012E+03	1.7418E+03	1.4964E+03	1.7287E+03	1.8904E+03
	Std	5.2322E+01	7.4516E+01	6.2601E+02	2.1994E+03	3.4170E+02	7.1460E+01	1.0656E+02	1.3680E+02	1.5074E+02	2.4726E+02
F12	Best	1.6262E+05	4.0213E+06	1.2047E+09	4.0234E+08	2.2107E+07	2.0031E+06	6.8412E+05	8.5532E+05	7.7370E+07	5.9556E+06
	Mean	5.7692E+06	2.7218E+07	2.0328E+09	9.6952E+08	3.7227E+08	6.2400E+06	4.2419E+06	5.7091E+06	3.1533E+08	2.2375E+07
	Std	4.6743E+06	1.9095E+07	4.0748E+08	3.6970E+08	3.4082E+08	3.1370E+06	2.8463E+06	5.4459E+06	1.5261E+08	1.3337E+07
F13	Best	5.7030E+03	1.3468E+04	2.0973E+08	6.3440E+06	7.3659E+04	3.0417E+03	6.5961E+03	2.8226E+03	3.8572E+06	3.3551E+04
	Mean	2.1805E+04	4.4932E+04	3.9510E+08	3.6781E+07	2.1890E+07	9.1735E+03	1.5998E+04	1.1332E+04	1.8464E+07	2.3006E+05
	Std	1.1430E+04	2.5659E+04	1.0329E+08	2.3831E+07	3.3136E+07	6.1190E+03	9.4381E+03	7.7613E+03	1.2079E+07	2.2370E+05
F14	Best	1.5360E+03	1.9063E+03	1.9055E+05	2.1165E+04	1.2970E+04	1.5200E+04	1.5649E+04	2.1553E+03	5.2986E+04	3.8584E+04
	Mean	1.8077E+03	6.5826E+04	6.4809E+05	1.6101E+05	5.7000E+05	7.6210E+04	7.1461E+04	2.9783E+04	4.6576E+05	2.3939E+05
	Std	1.8956E+02	5.5929E+04	3.1268E+05	9.8685E+04	5.7977E+05	4.3757E+04	4.7562E+04	3.3252E+04	3.8850E+05	1.6627E+05
F15	Best	2.1615E+03	3.5936E+03	8.4871E+06	2.2841E+04	2.6372E+04	1.8441E+03	2.3422E+03	2.0106E+03	1.7056E+05	2.5278E+03
	Mean	6.4379E+03	2.2461E+04	2.6119E+07	4.4362E+05	2.5828E+06	9.3681E+03	1.0743E+04	8.7553E+03	2.8011E+06	2.6350E+04
	Std	3.9671E+03	1.4666E+04	1.3965E+07	5.7830E+05	7.7912E+06	6.2748E+03	7.7850E+03	6.5189E+03	2.1062E+06	2.5135E+04
F16	Best	2.1354E+03	2.9226E+03	3.7299E+03	2.7202E+03	2.8484E+03	2.6576E+03	2.5314E+03	3.6980E+03	2.6821E+03	3.3207E+03
	Mean	3.0779E+03	3.6648E+03	4.5635E+03	3.2883E+03	3.7882E+03	3.2520E+03	3.2719E+03	4.5149E+03	3.7340E+03	3.9394E+03
	Std	3.4244E+02	3.9041E+02	3.5518E+02	3.0553E+02	4.5251E+02	3.9062E+02	4.5172E+02	3.2194E+02	4.9631E+02	3.5738E+02
F17	Best	2.3775E+03	2.6270E+03	2.7748E+03	2.3838E+03	3.0036E+03	2.2286E+03	2.1686E+03	2.0017E+03	2.4424E+03	2.8230E+03
	Mean	2.7994E+03	3.3434E+03	3.8192E+03	2.9952E+03	3.6013E+03	3.0171E+03	2.8593E+03	3.1160E+03	3.1397E+03	3.4096E+03
	Std	2.4619E+02	2.9163E+02	2.8520E+02	2.7802E+02	3.3359E+02	2.5309E+02	3.3731E+02	5.1167E+02	3.8048E+02	2.2994E+02
F18	Best	3.7954E+03	7.3458E+04	1.4138E+06	1.2752E+05	6.2889E+05	1.4027E+05	1.0878E+05	4.9158E+04	1.9874E+05	3.8207E+05
	Mean	5.8613E+04	5.9098E+05	6.0765E+06	1.4581E+06	3.5497E+06	8.5259E+05	8.6838E+05	3.7656E+05	4.5622E+06	2.7442E+06
	Std	7.7921E+04	5.5627E+05	4.3362E+06	1.0569E+06	3.5171E+06	6.1322E+05	5.1368E+05	3.1220E+05	3.2993E+06	1.5239E+06
F19	Best	2.2174E+03	6.1925E+03	5.6395E+06	3.2375E+04	1.7300E+04	2.8185E+03	2.1579E+03	2.0688E+03	1.3366E+05	7.5472E+03
	Mean	7.3595E+03	2.5497E+04	2.4370E+07	1.6139E+05	1.7929E+06	1.4697E+04	1.6694E+04	1.2260E+04	1.3489E+06	1.9150E+04
	Std	3.9523E+03	8.1732E+03	1.0652E+07	9.5140E+04	2.7954E+06	9.0050E+03	9.7398E+03	1.0314E+04	1.2465E+06	9.5367E+03
F20	Best	2.5180E+03	2.3742E+03	2.7325E+03	2.4865E+03	2.7722E+03	2.5964E+03	2.5909E+03	3.5232E+03	2.5817E+03	2.7475E+03
	Mean	2.8774E+03	3.0968E+03	3.2717E+03	2.9780E+03	3.3338E+03	3.0316E+03	3.3161E+03	3.8074E+03	3.0464E+03	3.2122E+03
	Std	1.9984E+02	3.3144E+02	2.6393E+02	2.0589E+02	2.5965E+02	3.0114E+02	3.6145E+02	1.3175E+02	3.0076E+02	2.6898E+02
F21	Best	2.3680E+03	2.4905E+03	2.6925E+03	2.5781E+03	2.5768E+03	2.4393E+03	2.3761E+03	2.6140E+03	2.5560E+03	2.5637E+03
	Mean	2.4631E+03	2.5907E+03	2.7319E+03	2.6195E+03	2.6802E+03	2.5244E+03	2.4385E+03	2.6626E+03	2.6239E+03	2.6544E+03
	Std	4.3196E+01	5.2773E+01	1.9844E+01	2.1787E+01	5.7542E+01	4.4399E+01	3.5296E+01	1.8541E+01	3.9873E+01	4.8311E+01
F22	Best	6.7987E+03	2.3447E+03	3.2003E+03	5.4288E+03	4.8107E+03	2.3313E+03	2.3089E+03	2.5429E+03	9.4390E+03	7.0214E+03
	Mean	1.0087E+04	1.0069E+04	7.6308E+03	9.4152E+03	1.1526E+04	9.7564E+03	1.3245E+04	1.4771E+04	1.1140E+04	8.8594E+03
	Std	1.1371E+03	1.8858E+03	5.8083E+03	2.4111E+03	2.5523E+03	1.8027E+03	3.9421E+03	2.8428E+03	9.5129E+02	8.4583E+02
F23	Best	2.8479E+03	3.0072E+03	3.1786E+03	3.0237E+03	3.0416E+03	2.9000E+03	2.8393E+03	3.0404E+03	2.9939E+03	3.0919E+03
	Mean	2.9338E+03	3.1453E+03	3.2088E+03	3.1393E+03	3.1748E+03	3.0500E+03	2.8932E+03	3.0965E+03	3.0824E+03	3.2264E+03
	Std	4.7904E+01	8.5304E+01	2.0915E+01	3.8963E+01	6.1739E+01	9.6164E+01	3.6843E+01	2.1783E+01	4.2069E+01	7.4182E+01
F24	Best	3.0125E+03	3.1681E+03	3.3146E+03	3.2174E+03	3.1874E+03	3.1031E+03	2.9912E+03	3.2323E+03	3.2070E+03	3.2221E+03
	Mean	3.0902E+03	3.3262E+03	3.3532E+03	3.2983E+03	3.3282E+03	3.2142E+03	3.0572E+03	3.2651E+03	3.2928E+03	3.4096E+03
	Std	4.8379E+01	1.2271E+02	1.5142E+01	3.8169E+01	7.5584E+01	7.5636E+01	4.6080E+01	1.4946E+01	5.5279E+01	7.1777E+01
F25	Best	3.0322E+03	3.0574E+03	3.6429E+03	3.7371E+03	3.0755E+03	3.0424E+03	3.0377E+03	3.0448E+03	3.2080E+03	2.9806E+03
	Mean	3.1110E+03	3.1584E+03	3.8005E+03	4.2779E+03	3.2609E+03	3.1456E+03	3.1056E+03	3.0990E+03	3.3671E+03	3.1301E+03
	Std	3.5737E+01	5.6819E+01	1.1913E+02	2.9234E+02	9.5714E+01	4.1801E+01	2.5326E+01	2.5660E+01	8.5678E+01	4.5933E+01
F26	Best	2.9860E+03	3.6398E+03	8.0625E+03	4.8993E+03	3.8068E+03	3.0963E+03	4.8921E+03	6.9757E+03	6.8200E+03	4.6792E+03
	Mean	5.5483E+03	7.7332E+03	8.5635E+03	7.4666E+03	8.0673E+03	6.3767E+03	5.5010E+03	7.2866E+03	7.6992E+03	7.5752E+03
	Std	9.8548E+02	1.3803E+03	2.1426E+02	1.1438E+03	1.2007E+03	2.0040E+03	4.8579E+02	1.6993E+02	5.7225E+02	1.0166E+03
F27	Best	3.2660E+03	3.3398E+03	3.4380E+03	3.5595E+03	3.3393E+03	3.4570E+03	3.2864E+03	3.2629E+03	3.3605E+03	3.5924E+03
	Mean	3.4936E+03	3.5701E+03	3.5116E+03	3.7629E+03	3.5513E+03	3.6681E+03	3.4236E+03	3.3481E+03	3.4969E+03	3.7463E+03
	Std	1.3416E+02	1.5752E+02	5.1577E+01	9.1196E+01	1.1520E+02	1.3683E+02	9.7737E+01	6.4026E+01	9.0940E+01	1.1915E+02
F28	Best	3.2761E+03	3.3520E+03	3.7543E+03	4.3455E+03	3.3636E+03	3.3500E+03	3.3137E+03	3.2745E+03	3.3966E+03	3.3281E+03
	Mean	3.3613E+03	3.5025E+03	3.9405E+03	4.8506E+03	4.2937E+03	3.4423E+03	3.3817E+03	3.3499E+03	3.6407E+03	3.4142E+03
	Std	4.1062E+01	9.0431E+01	1.3177E+02	2.9620E+02	1.3669E+03	5.7332E+01	3.5195E+01	3.6448E+01	1.0751E+02	5.5563E+01
F29	Best	3.5775E+03	4.0253E+03	4.5959E+03	4.6803E+03	4.3264E+03	3.7351E+03	3.5819E+03	4.1589E+03	3.6850E+03	4.4702E+03
	Mean	4.2611E+03	4.6943E+03	5.5336E+03	5.4406E+03	4.8183E+03	4.4037E+03	4.2100E+03	4.7933E+03	4.4121E+03	5.0798E+03
	Std	3.4768E+02	3.8582E+02	3.3499E+02	4.1664E+02	3.4596E+02	3.0366E+02	2.7398E+02	3.3744E+02	3.5888E+02	3.3495E+02
F30	Best	7.8209E+05	1.3797E+06	6.8969E+07	3.0117E+07	2.4684E+06	2.3959E+06	8.6368E+05	1.1290E+06	5.6045E+06	2.7514E+06
	Mean	1.4393E+06	3.7645E+06	1.3771E+08	5.5311E+07	1.2973E+07	4.3730E+06	1.3741E+06	1.9651E+06	1.4093E+07	4.2771E+06
	Std	6.2791E+05	2.4260E+06	3.7086E+07	1.7702E+07	8.0992E+06	1.3638E+06	3.7860E+05	9.6758E+05	6.0105E+06	1.1205E+06

**Table A4:** Results of CEC2017 100D functions obtained by EDECO and its competitors.

Function	Index	EDECO	ECO	SAO	CFOA	DBO	MRFO	ISGTOA	EMTLBO	TERIME	AFDB-ARO
F1	Best	6.4093E+07	6.0811E+08	3.5632E+10	5.4419E+10	9.0795E+09	4.6751E+08	9.9740E+07	9.1571E+07	2.1991E+10	4.5955E+06
	Mean	1.4361E+08	1.6215E+09	4.6462E+10	6.6144E+10	4.0977E+10	8.9030E+08	2.6073E+08	1.6669E+08	3.1931E+10	1.1789E+07
	Std	7.8703E+07	7.4915E+08	4.2203E+09	6.0628E+09	3.1955E+10	4.4942E+08	1.5579E+08	5.2449E+07	5.9902E+09	5.8211E+06
F3	Best	4.0463E+04	6.6301E+04	2.4806E+05	1.5671E+05	2.8620E+05	1.5744E+05	1.9993E+05	1.3521E+05	2.4960E+05	3.1429E+05
	Mean	6.1024E+04	1.1645E+05	2.8120E+05	2.0870E+05	3.6571E+05	2.0055E+05	2.2948E+05	2.0759E+05	3.4402E+05	3.3288E+05
	Std	1.3122E+04	2.5409E+04	1.4935E+04	2.1433E+04	5.1842E+04	2.0904E+04	1.1920E+04	4.8423E+04	4.8397E+04	1.0476E+04
F4	Best	7.6212E+02	9.4142E+02	4.5433E+03	5.4287E+03	1.5645E+03	1.0202E+03	7.8019E+02	7.9924E+02	2.2109E+03	7.2445E+02
	Mean	8.8858E+02	1.1599E+03	5.5153E+03	8.4545E+03	2.9785E+03	1.1847E+03	9.2100E+02	9.2000E+02	3.3919E+03	8.2946E+02
	Std	7.3712E+01	1.6728E+02	6.7475E+02	1.2564E+03	1.5163E+03	1.4424E+02	7.8512E+01	6.5828E+01	5.8852E+02	4.5782E+01
F5	Best	8.5002E+02	1.0185E+03	1.5643E+03	1.3647E+03	1.2312E+03	1.1118E+03	8.4750E+02	1.3914E+03	1.2981E+03	1.0625E+03
	Mean	1.0275E+03	1.2708E+03	1.6158E+03	1.4657E+03	1.5057E+03	1.2595E+03	9.3635E+02	1.4372E+03	1.4114E+03	1.2890E+03
	Std	1.0774E+02	1.2176E+02	2.3377E+01	4.1069E+01	1.2239E+02	7.9360E+01	6.5141E+01	2.8196E+01	6.6943E+01	9.7417E+01
F6	Best	6.1763E+02	6.4768E+02	6.4894E+02	6.5933E+02	6.4245E+02	6.3969E+02	6.1626E+02	6.0931E+02	6.2868E+02	6.0570E+02
	Mean	6.3915E+02	6.6007E+02	6.5363E+02	6.6782E+02	6.6146E+02	6.5400E+02	6.2490E+02	6.1172E+02	6.4324E+02	6.1032E+02
	Std	8.2945E+00	6.1166E+00	2.8516E+00	3.7147E+00	8.0898E+00	7.5418E+00	5.3530E+00	1.3589E+00	7.1420E+00	2.4438E+00
F7	Best	1.4940E+03	1.8708E+03	2.2571E+03	2.0971E+03	1.5914E+03	1.8735E+03	1.3469E+03	1.6680E+03	2.2462E+03	1.5950E+03
	Mean	1.8011E+03	2.4316E+03	2.3744E+03	2.4179E+03	2.0263E+03	2.2564E+03	1.4935E+03	1.7402E+03	2.6407E+03	1.8356E+03
	Std	1.9682E+02	2.4183E+02	6.2421E+01	1.1261E+02	1.6821E+02	2.8758E+02	1.1602E+02	3.0045E+01	2.3334E+02	1.3959E+02
F8	Best	1.1644E+03	1.3980E+03	1.8549E+03	1.7396E+03	1.5839E+03	1.3472E+03	1.1418E+03	1.6358E+03	1.5582E+03	1.3971E+03
	Mean	1.3591E+03	1.6245E+03	1.9207E+03	1.8359E+03	1.8703E+03	1.5863E+03	1.2519E+03	1.7240E+03	1.7214E+03	1.6172E+03
	Std	9.8046E+01	1.1160E+02	3.1367E+01	5.1949E+01	1.2464E+02	1.1414E+02	1.0182E+02	4.0498E+01	9.5055E+01	9.1618E+01
F9	Best	6.4841E+03	2.0204E+04	2.4678E+04	2.8417E+04	2.7954E+04	2.5934E+04	7.3478E+03	1.9319E+03	2.5362E+04	2.0557E+04
	Mean	1.6538E+04	2.7902E+04	3.5242E+04	3.3004E+04	6.2106E+04	3.3334E+04	1.5419E+04	3.5161E+03	4.1077E+04	3.0685E+04
	Std	6.5364E+03	4.3580E+03	5.5667E+03	3.1879E+03	1.3189E+04	6.8115E+03	4.0988E+03	1.8623E+03	1.1121E+04	4.5494E+03
F10	Best	1.4943E+04	1.4207E+04	2.9421E+04	2.0445E+04	1.5559E+04	1.2625E+04	2.4677E+04	2.9213E+04	1.9660E+04	1.3310E+04
	Mean	1.8405E+04	1.8867E+04	3.0418E+04	2.4330E+04	2.2372E+04	1.7040E+04	2.9320E+04	3.0748E+04	2.2291E+04	1.4707E+04
	Std	2.4196E+03	2.5409E+03	4.9822E+02	1.3768E+03	6.1053E+03	2.4499E+03	1.9333E+03	4.6133E+02	1.3058E+03	9.0095E+02
F11	Best	2.6776E+03	3.1419E+03	3.1504E+04	2.7488E+04	4.3360E+04	1.4914E+04	1.8090E+04	4.1607E+03	1.1006E+04	3.5813E+04
	Mean	3.1285E+03	5.3797E+03	4.7227E+04	4.6798E+04	6.9393E+04	2.3603E+04	2.8114E+04	1.2780E+04	2.3087E+04	6.4962E+04
	Std	3.3494E+02	1.3946E+03	7.2536E+03	8.1994E+03	2.2234E+04	4.2792E+03	4.9756E+03	8.1886E+03	8.5232E+03	1.6572E+04
F12	Best	1.3646E+07	7.1724E+07	8.6900E+09	6.2214E+09	7.8804E+08	3.1173E+07	1.2097E+07	1.7337E+07	1.5451E+09	3.3473E+07
	Mean	4.8815E+07	2.3802E+08	1.2173E+10	9.7865E+09	2.3839E+09	8.7579E+07	4.1293E+07	4.3574E+07	3.4930E+09	1.0621E+08
	Std	2.5076E+07	1.0824E+08	1.4290E+09	2.0611E+09	8.7968E+08	3.6325E+07	1.7645E+07	2.0526E+07	9.9177E+08	4.1603E+07
F13	Best	6.4394E+03	2.6385E+04	9.1054E+08	9.1188E+07	2.2712E+05	1.0386E+04	1.4118E+04	6.6219E+03	3.5726E+07	1.4415E+04
	Mean	1.3939E+04	9.2609E+04	1.3446E+09	5.8024E+08	7.5769E+07	1.8477E+04	2.3862E+04	1.1945E+04	8.8798E+07	4.2557E+05
	Std	9.9074E+03	6.6426E+04	2.4487E+08	2.2919E+08	6.8310E+07	8.0874E+03	5.7207E+03	3.8850E+03	5.2428E+07	3.4788E+05
F14	Best	3.2207E+03	9.8429E+04	2.8928E+06	3.6465E+05	9.0888E+05	2.7660E+05	2.9125E+05	7.0233E+04	2.1120E+06	2.5136E+05
	Mean	6.5823E+04	5.3485E+05	5.4714E+06	2.6675E+06	4.7113E+06	8.5821E+05	9.4091E+05	4.1067E+05	8.4961E+06	2.4644E+06
	Std	5.3852E+04	2.7186E+05	1.4656E+06	9.4721E+05	3.0285E+06	4.9473E+05	3.7929E+05	3.0308E+05	4.4363E+06	1.4171E+06
F15	Best	2.3150E+03	4.8222E+03	2.3791E+08	3.8192E+06	6.4001E+04	2.4095E+03	4.7605E+03	2.4786E+03	2.8956E+06	1.4789E+04
	Mean	7.2317E+03	2.5925E+04	3.4627E+08	3.5469E+07	4.5170E+06	4.9756E+03	8.6631E+03	6.4260E+03	1.1330E+07	7.5247E+04
	Std	5.5274E+03	1.6154E+04	7.0196E+07	2.4602E+07	9.8311E+06	3.2721E+03	4.2534E+03	6.0441E+03	7.1382E+06	5.1243E+04
F16	Best	4.7424E+03	4.7474E+03	9.7048E+03	6.5083E+03	5.3172E+03	4.6752E+03	3.6894E+03	7.4613E+03	5.3675E+03	4.9856E+03
	Mean	5.5830E+03	6.3978E+03	1.0476E+04	7.9632E+03	7.7569E+03	5.8261E+03	5.3651E+03	8.6555E+03	7.4023E+03	6.3867E+03
	Std	5.6509E+02	7.0682E+02	3.7297E+02	6.1990E+02	8.7524E+02	6.4629E+02	8.0469E+02	5.5024E+02	8.2011E+02	5.3963E+02
F17	Best	3.8551E+03	4.5805E+03	7.3927E+03	4.8945E+03	5.6912E+03	4.2075E+03	3.3422E+03	3.2757E+03	5.0000E+03	5.1547E+03
	Mean	4.5337E+03	5.6151E+03	8.1467E+03	5.7177E+03	6.9405E+03	4.8591E+03	4.6841E+03	4.9468E+03	5.5768E+03	5.8943E+03
	Std	4.2414E+02	5.3745E+02	3.8130E+02	4.4822E+02	6.6657E+02	4.7794E+02	5.8098E+02	1.0638E+03	4.5075E+02	4.5471E+02
F18	Best	4.3418E+04	3.0406E+05	5.5141E+06	1.1565E+06	1.5655E+06	4.1188E+05	4.5790E+05	2.2056E+05	3.7643E+06	1.4942E+06
	Mean	1.4338E+05	9.0488E+05	1.2158E+07	2.2582E+06	8.5901E+06	1.4103E+06	1.8148E+06	6.2667E+05	1.5943E+07	3.4406E+06
	Std	7.3365E+04	7.7698E+05	4.9804E+06	6.0869E+05	3.3367E+06	6.0317E+05	1.2761E+06	4.9173E+05	8.2148E+06	1.4594E+06
F19	Best	2.1407E+03	8.1980E+03	1.9383E+08	7.9498E+06	1.6328E+06	2.3924E+03	2.3973E+03	2.2590E+03	3.3597E+06	7.1631E+03
	Mean	5.0396E+03	6.0871E+04	3.3563E+08	3.8904E+07	2.2604E+07	6.0791E+03	7.5209E+03	6.7378E+03	1.4327E+07	1.1509E+05
	Std	2.9285E+03	8.0844E+04	7.4788E+07	2.3203E+07	2.5585E+07	5.1265E+03	5.7939E+03	6.4609E+03	7.1633E+06	1.2569E+05
F20	Best	3.7956E+03	4.1226E+03	4.8723E+03	4.3659E+03	4.3748E+03	4.1083E+03	3.6959E+03	6.5424E+03	4.1070E+03	4.3926E+03
	Mean	4.9623E+03	5.1888E+03	6.5603E+03	5.2178E+03	5.7324E+03	4.9617E+03	6.2439E+03	7.0705E+03	5.2824E+03	5.1608E+03
	Std	3.7074E+02	6.2052E+02	5.6847E+02	3.9827E+02	7.2338E+02	5.2654E+02	7.2337E+02	2.0211E+02	5.0263E+02	3.8231E+02
F21	Best	2.6194E+03	2.9237E+03	3.3190E+03	3.1841E+03	3.3325E+03	2.7405E+03	2.6411E+03	3.1862E+03	3.1202E+03	3.0421E+03
	Mean	2.8083E+03	3.2018E+03	3.4089E+03	3.2673E+03	3.5474E+03	3.0409E+03	2.7383E+03	3.2268E+03	3.2604E+03	3.1906E+03
	Std	1.0277E+02	1.5398E+02	3.2660E+01	5.3679E+01	9.9253E+01	1.1397E+02	6.9729E+01	2.1495E+01	7.0566E+01	8.3527E+01
F22	Best	1.7614E+04	1.9499E+04	7.4025E+03	1.6604E+04	1.8554E+04	1.7447E+04	2.4826E+04	3.0568E+04	2.1620E+04	1.5437E+04
	Mean	2.2725E+04	2.2688E+04	2.4609E+04	2.7098E+04	2.2372E+04	2.0620E+04	3.1464E+04	3.2649E+04	2.4806E+04	1.7425E+04
	Std	2.4022E+03	1.9610E+03	1.1631E+04	2.4908E+03	4.2882E+03	1.5848E+03	2.1144E+03	6.3771E+02	1.3441E+03	1.1396E+03
F23	Best	3.1682E+03	3.5303E+03	3.9090E+03	3.8556E+03	3.7089E+03	3.4956E+03	3.1559E+03	3.4709E+03	3.5583E+03	3.4100E+03
	Mean	3.3771E+03	3.8586E+03	4.0133E+03	3.9695E+03	3.9365E+03	3.6897E+03	3.3083E+03	3.6690E+03	3.6609E+03	3.5250E+03
	Std	1.1487E+02	1.7642E+02	4.4756E+01	6.8572E+01	1.1981E+02	1.0000E+02	8.1744E+01	5.8575E+01	7.5736E+01	7.5286E+01
F24	Best	3.7263E+03	4.0586E+03	4.4380E+03	4.4667E+03	4.2727E+03	4.2481E+03	3.5457E+03	4.0783E+03	4.1346E+03	4.2543E+03
	Mean	3.9355E+03	4.6301E+03	4.5658E+03	4.6656E+03	4.6140E+03	4.5428E+03	3.7692E+03	4.1699E+03	4.3724E+03	4.4724E+03
	Std	1.2929E+02	2.7836E+02	6.9515E+01	8.8737E+01	1.7135E+02	1.8692E+02	9.3922E+01	4.6972E+01	9.7893E+01	1.1071E+02
F25	Best	3.4223E+03	3.6827E+03	5.7920E+03	6.9568E+03	4.0796E+03	3.6645E+03	3.4618E+03	3.4474E+03	4.9998E+03	3.3405E+03
	Mean	3.5806E+03	3.9365E+03	6.9471E+03	7.9971E+03	6.2492E+03	3.8484E+03	3.6179E+03	3.6168E+03	6.0505E+03	3.4439E+03
	Std	7.9968E+01	1.8347E+02	4.8499E+02	5.6848E+02	2.7327E+03	8.8690E+01	7.1202E+01	6.1172E+01	6.5654E+02	5.5448E+01
F26	Best	1.0533E+04	8.5487E+03	1.7934E+04	1.8823E+04	1.5170E+04	1.3860E+04	9.7523E+03	1.2915E+04	1.4236E+04	1.1040E+04
	Mean	1.2744E+04	1.7401E+04	1.8936E+04	2.1379E+04	1.9009E+04	1.9604E+04	1.1189E+04	1.4550E+04	1.6705E+04	1.7907E+04
	Std	1.3238E+03	3.0059E+03	4.2890E+02	2.1401E+03	1.9271E+03	3.1054E+03	1.0519E+03	5.6074E+02	8.9228E+02	2.2523E+03
F27	Best	3.5303E+03	3.5501E+03	3.8565E+03	4.2956E+03	3.5366E+03	3.7935E+03	3.5249E+03	3.4047E+03	3.6293E+03	3.6656E+03
	Mean	3.6733E+03	3.8081E+03	4.1141E+03	4.6228E+03	3.7682E+03	4.0820E+03	3.6646E+03	3.5011E+03	3.8731E+03	3.8970E+03
	Std	1.0819E+02	1.4572E+02	1.4381E+02	1.7352E+02	1.6798E+02	1.7284E+02	1.0799E+02	5.7106E+01	1.0054E+02	1.0671E+02
F28	Best	3.5166E+03	3.7497E+03	7.1696E+03	9.4069E+03	5.5837E+03	3.8265E+03	3.6324E+03	3.5310E+03	5.4898E+03	3.4881E+03
	Mean	3.6374E+03	4.5645E+03	8.0727E+03	1.0781E+04	1.5105E+04	4.2162E+03	3.7474E+03	3.6600E+03	6.8604E+03	4.1791E+03
	Std	6.4295E+01	2.3610E+03	5.7234E+02	7.4127E+02	4.5069E+03	2.5532E+02	6.8253E+01	5.8586E+01	1.2241E+03	2.3644E+03
F29	Best	5.9190E+03	6.9693E+03	1.0007E+04	9.5806E+03	7.1834E+03	5.9217E+03	5.8411E+03	7.4655E+03	6.2682E+03	7.1984E+03
	Mean	6.7814E+03	7.9910E+03	1.1244E+04	1.0430E+04	8.7717E+03	7.3753E+03	6.9994E+03	8.6778E+03	7.6190E+03	7.8121E+03
	Std	6.8647E+02	5.8233E+02	4.4027E+02	4.6823E+02	8.8805E+02	5.9343E+02	6.5606E+02	5.8396E+02	5.4083E+02	4.2690E+02
F30	Best	3.2533E+04	5.1093E+05	8.6458E+08	2.3485E+08	7.6644E+06	1.6746E+05	1.3255E+05	7.1824E+04	1.3486E+07	1.7498E+05
	Mean	9.0560E+04	3.9727E+06	1.1898E+09	4.6489E+08	4.7662E+07	4.2200E+05	4.8348E+05	2.6844E+05	6.2636E+07	5.5747E+05
	Std	6.0173E+04	2.7152E+06	1.8612E+08	1.6817E+08	3.1427E+07	2.2887E+05	2.6316E+05	1.2535E+05	2.9629E+07	2.1458E+05
